# Crosstalk between Lipid Metabolism and Bone Homeostasis: Exploring Intricate Signaling Relationships

**DOI:** 10.34133/research.0447

**Published:** 2024-08-20

**Authors:** Haixiang Xiao, Wenming Li, Yi Qin, Zhixiang Lin, Chen Qian, Mingzhou Wu, Yu Xia, Jiaxiang Bai, Dechun Geng

**Affiliations:** ^1^ Department of Orthopedics, The First Affiliated Hospital of Soochow University, Suzhou, Jiangsu 215006, China.; ^2^ Department of Orthopedics, Centre for Leading Medicine and Advanced Technologies of IHM, The First Affiliated Hospital of USTC, Division of Life Sciences and Medicine, University of Science and Technology of China, Hefei 230022, China.; ^3^ Department of Orthopedics, Jingjiang People’s Hospital Affiliated to Yangzhou University, Jingjiang 214500, Jiangsu Province, China.

## Abstract

Bone is a dynamic tissue reshaped by constant bone formation and bone resorption to maintain its function. The skeletal system accounts for approximately 70% of the total volume of the body, and continuous bone remodeling requires quantities of energy and material consumption. Adipose tissue is the main energy storehouse of the body and has a strong adaptive capacity to participate in the regulation of various physiological processes. Considering that obesity and metabolic syndrome have become major public health challenges, while osteoporosis and osteoporotic fractures have become other major health problems in the aging population, it would be interesting to explore these 2 diseases together. Currently, an increasing number of researchers are focusing on the interactions between multiple tissue systems, i.e., multiple organs and tissues that are functionally coordinated together and pathologically pathologically interact with each other in the body. However, there is lack of detailed reviews summarizing the effects of lipid metabolism on bone homeostasis and the interactions between adipose tissue and bone tissue. This review provides a detailed summary of recent advances in understanding how lipid molecules and adipose-derived hormones affect bone homeostasis, how bone tissue, as a metabolic organ, affects lipid metabolism, and how lipid metabolism is regulated by bone-derived cytokines.

## Introduction

Over the past decades, the seemingly unrelated fields of lipid metabolism and bone homeostasis have become popular research topics, with the development of interdisciplinary studies focusing on the interactions between human tissues, organs, and systems. Particularly, in recent years, a large number of studies, including both preclinical and clinical studies, have revealed complex and profound links between lipid metabolism and bone homeostasis. The bone marrow niche, which is composed of bone marrow mesenchymal stem cells (BMSCs), osteoblasts, monocytes, macrophages, osteoclasts, osteoblasts, adipocytes, and others [1], fully reflects the close relationship between bone and adipose tissue metabolism.

On the one hand, BMSCs are subject to multiple influences from adipocytes and osteoclasts in their choice between osteogenic and adipogenic differentiation [2], e.g., fatty acids, leptin, adiponectin, and other factors released from adipocytes alter the fate of BMSCs [3,4]. On the other hand, osteoclast differentiation is closely related to osteoblasts and adipocytes and is regulated by feedback from receptor activator of nuclear factor-κB (NF-κB) ligand (RANKL) and osteoprotegerin (OPG) secreted by osteoblasts. Furthermore, the type and proportion of lipids undergo significant changes during the fusion process of osteoclasts [5]. Therefore, adipogenesis, osteogenesis, and osteoclastogenesis are constantly interconnected, regulated, and influenced by each other.

Disruption of metabolic homeostasis in any cell line may lead to disruption of lipid metabolism and bone homeostasis, resulting in concurrent disorders of lipid metabolism and imbalances in bone metabolism [6,7]. However, most of the existing studies have focused on fundamental research with inconsistent and, in some cases, contradictory conclusions, and no consensus has been reached, especially with regard to key molecular mechanisms, signaling pathways, and important clinical findings.

Therefore, it would be interesting to coordinate and summarize the multiple perspectives based on the latest available literature and provide some suggestions for bone–adipose tissue crosstalk studies. Considering the rapid socioeconomic development and improvement of people’s living standards over the past half century, disorders of lipid metabolism, dyslipidemia, obesity, and metabolic syndrome have become major public health challenges for both developing and developed countries [8,9]. Moreover, the aging of the population has made osteoporosis and osteoporotic fractures major global health issues [10,11]. These 2 common diseases have prompted further understanding of the relationship between lipid metabolism and bone homeostasis, and it will be important to elucidate the molecular signaling or cellular mechanisms that underlie the intrinsic link between these 2 metabolic pathways.

In this review, we discussed the complex effects of lipid metabolism and several important metabolic products on bone homeostasis. Specifically, we summarized the effects of fatty acids, cholesterol, adiponectin, and leptin on the metabolism, differentiation, and function of osteoblasts and osteoclasts, as well as the signaling receptors and pathways involved. We also discuss the potential drug development and clinical applications of certain important signaling targets, which will be useful sources of inspiration for other readers and researchers. We have also completed a series of reviews on strategies for targeting skeletal disease [12,13]. Furthermore, we elucidated how the skeletal system, as a crucial metabolic and endocrine organ, influences lipid metabolism, and 3 bone-derived cytokines responsible for modulating lipid metabolism were individually investigated. Finally, based on the current research, we analyzed and provided insights into future research priorities and challenges from both basic research and clinical application perspectives.

## Lipid Metabolism Regulates Bone Homeostasis

Lipids are a heterogeneous class of organic molecules characterized by hydrophobicity and low molecular weight (commonly <1,000 dalton). The structure of lipids consists of linear alkyl chains, usually with an even number of carbon atoms, and may be saturated or unsaturated, featuring double bonds at characteristic positions. Additionally, lipids may contain isoprene units with either linear or cyclic structures. Lipid metabolism and its metabolites play a crucial role in modulating bone homeostasis through various mechanisms. First, lipids, as fundamental components of biological membranes in bone cells, determine the physicochemical properties of the membrane, thus influencing cellular behavior [14]. Second, lipid metabolism, particularly fatty acid β-oxidation, serves as a vital energy source for bone cells during energy-demanding processes such as osteoblast differentiation and osteoclast bone resorption, where glycolysis alone is insufficient [2,15]. Third, lipids or their metabolic intermediates act as signaling molecules binding to receptors on the surface of bone cells, thereby altering downstream signaling pathways and cellular behavior [16]. Moreover, adipose tissue or adipocytes produce cytokines that exert paracrine or endocrine effects on the differentiation potential, direction, and cellular function of bone precursor cells [17,18].

Therefore, imbalances in exogenous lipid intake or disruptions in endogenous lipid homeostasis can have either positive or negative effects on bone homeostasis. This section will explore the 2 significant lipid molecules, fatty acids and cholesterol, as well as 2 representative adipokines, leptin and adiponectin, and their distinct impacts on the biological behavior of osteoblastic and osteoclastic cell lineages, encompassing energy metabolism, membrane homeostasis, and cellular signaling. In addition, clinical studies investigating the influence of lipid metabolism on bone homeostasis and the potential translational applications of these findings will also be discussed.

### Fatty acids are involved in bone homeostasis

Fatty acids are the fundamental components of lipids, and both the de novo synthesis and β-oxidation of fatty acids are essential processes in lipid metabolism. Therefore, the effects of fatty acids on the metabolism, differentiation, and function of bone cells are diverse. Fatty acids may alter the biological membrane stability, energy metabolism, and intracellular signal transduction of bone cells. This regulatory function of fatty acids may occur through direct actions on bone cells or by linking lipid metabolism and bone metabolism via a crucial hub, namely, vascular endothelial cells, particularly the H-type vessels discovered in recent years. These vessels modify the energy metabolism and signal transduction of bone cells, thereby indirectly regulating bone homeostasis.

#### The sources and metabolic pathways of fatty acids in bone tissue

**Fig. 1.**
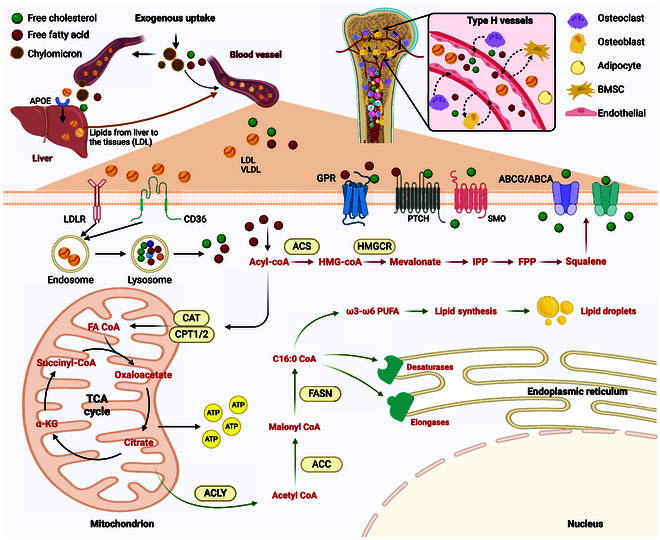
The metabolic processes of fatty acids and cholesterol. Exogenous intake of lipids from food, forming lipoprotein particles. Lipoprotein releases free fatty acids and cholesterol for tissue uptake and utilization. G protein-coupled receptor (GPR) and PTCH receptors on the cell membrane can bind to free fatty acids and free cholesterol, initiating signaling pathways and regulating osteogenesis and osteoclastogenesis. Free fatty acids are converted into fatty acyl-CoA by the acyl-CoA synthetase (ACS) family and transported to the mitochondrial matrix by carnitine palmitoyltransferase (CPT) and carnitine-acylcarnitine translocase (CAT), initiating the β-oxidation pathway of fatty acids and generating ATP for energy supply. One of the products of the tricarboxylic acid cycle, citrate, is cleaved by ATP citrate lyase (ACLY) to produce acetyl-CoA, which initiates the de novo synthesis of fatty acids. Acetyl-CoA carboxylase (ACC) catalyzes the conversion of acetyl-CoA to malonyl-CoA. Through elongation by fatty acid synthase (FASN), the primary product C16:0-CoA is formed, followed by catalysis by endoplasmic reticulum elongase and desaturases to generate fatty acids with different lengths and degrees of saturation. These fatty acids can further undergo mitochondrial β-oxidation or be stored as lipid droplets. In addition, acyl-CoA also serves as a substrate for cholesterol synthesis, and cholesterol can be effluxed from cells through ATP-binding cassette transporter A1 (ABCA1) and ATP-binding cassette transporter G1 (ABCG1). Type-H vessels are essential bone metabolism promoters during bone replacement and are closely connected to osteoblasts and osteoclasts.

In bone tissue, fatty acids primarily originate from 2 sources: exogenous dietary intake and endogenous de novo synthesis. Dietary lipids are broken down and processed in the intestine through a series of digestive enzymes and bile salts, after which triglycerides and cholesterol are packaged into chylomicrons within the intestinal epithelium. These chylomicrons are then absorbed via the lymphatic system [19] and enter the bloodstream. In capillaries, lipoprotein lipase breaks down a portion of the chylomicrons, releasing free fatty acids that are absorbed and utilized by target tissues such as adipose tissue and bone tissue [20] (Fig. 1). The remaining chylomicrons are cleared by the liver. As a sensor and regulatory hub for lipid homeostasis, the liver plays a critical role; hepatocytes take up chylomicron remnants through the cell membrane surface apolipoprotein E, and chylomicron remnants yield decomposition products, including free fatty acids and circulating glucose, which serve as substrates for de novo triglyceride synthesis.

Triglycerides, cholesterol, and other breakdown products of chylomicron remnants are repackaged into lipoprotein particles, such as very low-density lipoprotein (VLDL), low-density lipoprotein (LDL), and high-density lipoprotein (HDL), and released into circulation [21], where they encounter lipoprotein lipase and release free fatty acids. Endogenous fatty acid synthesis predominantly occurs in the cytoplasm and mitochondria of cells. Acetyl-CoA (coenzyme A), generated through processes such as glycolysis, amino acid metabolism, and ketone body metabolism in mitochondria, is transported to the cytoplasm. In the cytoplasm, acetyl-CoA carboxylase catalyzes the carboxylation of acetyl-CoA, forming malonyl-CoA. Subsequently, fatty acid synthase condenses 7 malonyl-CoA molecules with one acetyl-CoA molecule, producing palmitic acid (PA) ester as the initial product of fatty acid synthesis (Fig. 1). PA ester further undergoes elongation and desaturation, which are mediated by elongase and desaturases, respectively, resulting in the production of fatty acids with varying chain lengths and degrees of saturation.

The role of vascular endothelial cells in bone energy metabolism has received extensive attention in recent years [22]. Vessels not only supply bone tissue with essential nutrients, oxygen, and growth factors but also transport lipids and lipid metabolic products to bone cells (Fig. 1). Knowledge of the cellular communication mechanisms between vascular endothelial cells and the bone microenvironment has rapidly expanded recently. A subtype of capillary closely associated with osteoblasts is known as the H-type vessel, which is characterized by high expression of CD31 and endomucin, and is primarily located near the growth plate at the metaphysis and periosteum of the diaphysis [23]. The function of H-type vessels may be more than just transporting lipids or other nutrients. There is evidence of molecular crosstalk between osteoblastic and osteoclastic lineages and H-type endothelial cells. For instance, SLIT3 is an osteoblast-derived angiogenic factor, and knockout of the SLIT3 gene may impair H-type vessel formation, consequently leading to reduced bone mass [24].

In addition, H-vessel formation is an essential bone regeneration enhancer during bone deficiency repair. On the one hand, osteogenic lineage cells around H vessels had a higher bone formation capacity than other osteoblasts, and the expression levels of Osterix and Runx2 were also increased [25]. On the other hand, H vessels also produce various factors, such as vascular endothelial growth factor (VEGF) and Noggin, to facilitate osteogenic differentiation of osteogenic progenitor cells [25]. Although type H vessels are highly coupled with osteogenesis, the related mechanism has not been elucidated. Therefore, further studies are needed to elucidate the mechanism and to provide directions for the future treatment of bone diseases by targeting type H vessels.

#### Fatty acids affect cell membrane renewal in bone tissue

As crucial constituents involved in the construction of eukaryotic cell membranes, the type and composition ratio of fatty acids profoundly influence the chemical composition and physical properties of the cellular membrane, consequently altering the biological behavior of bone cells. Specifically, in osteoblast cell lineages, remodeling the lipidome of mesenchymal stem cells (MSCs) with dietary fat alters the specific membrane composition and biophysical properties of MSCs, ultimately altering their osteogenic differentiation potential. Supplementation with docosahexaenoic acid (DHA) induces extensive lipidomic changes in MSCs, mimicking the composition and structure of osteoblastic plasma membranes and thereby enhancing MSC osteogenic differentiation [26]. Analysis of lipid metabolism during osteogenic-induced differentiation of human adipose tissue stem cells using nuclear magnetic resonance spectroscopy revealed notable lipid alterations primarily between days 1 and 7, characterized by increased phospholipids and longer, more unsaturated fatty acids. A tendency toward lipidome stabilization was observed after day 7. This evidence indicates that during different stages of osteoblastic cell differentiation, the composition and proportions of fatty acids undergo dynamic changes, which may be associated with alterations in membrane fluidity during the differentiation process [27].

However, few studies have focused on investigating the effect of fatty acids on the phenotype of osteoclast cell membranes. It can be inferred that the effect of free fatty acids on the structure and composition of the cellular membrane is ubiquitous across human tissue cells, and mature osteoclasts and mononuclear macrophages are no exception. In future studies, it would be meaningful to investigate the impact of fatty acids on lipid rafts in osteoclasts, changes in membrane fluidity, and their effects on osteoclast fusion and resorptive function.

#### Fatty acid and energy metabolism in bone tissue cells

Since the discovery of fatty acids as substrates capable of providing energy to bone tissue and isolated cells by Fleisch and colleagues in 1987 [28], numerous in vivo and in vitro studies have verified that fatty acids are key substrates for energy metabolism in cell differentiation and bone remodeling. A recent high-quality study showed that the differential fate of BMSCs is driven by a decrease in extracellular lipids. Serum lipid deprivation prevents the differentiation of BMSCs toward osteoblasts, as evidenced by a decrease in collagen type I α 1 (Col I a1)-expressing cells and reduced mineralization. This process leads to slower fracture healing, which is reversed by fatty acid supplementation [15]. The above phenomenon indicates that relying solely on glucose as a substrate for energy metabolism may not be sufficient to meet the rapidly increasing energy demands of skeletal progenitor cells during differentiation.

When the energy demand of bone cells increases, stored lipids can be rapidly mobilized and broken down through lipolysis [29]. Lipolysis involves cytoplasmic lipases acting on lipid droplets in 3 steps: (a) adipose triglyceride lipase hydrolyzes triglycerides, releasing a fatty acid and producing diacylglycerol; (b) hormone-sensitive lipase converts diacylglycerol to monoacylglycerol, releasing another fatty acid; and (c) monoacylglycerol lipase hydrolyzes monoacylglycerol, producing glycerol and a final free fatty acid. The resulting free fatty acids can be utilized by the cell or neighboring cells for energy production.

In bone tissue, bone cells may take up fatty acids in the form of free fatty acids or as part of lipoprotein complexes (Fig. 1). Various forms of G protein-coupled receptors (GPRs) on the cell membrane transport free fatty acids directly into the cell. Cell membrane receptors such as LDL receptor (LDLR) and cluster of differentiation 36 (CD36) transport lipoprotein complexes into the cell through endocytosis or other mechanisms. Subsequently, lipoproteins may be released within the cell through lysosomal degradation, leading to the presence of free fatty acids [30]. Compared with wild-type mice, knockout CD36 mice exhibit a low bone mass phenotype, and in vitro cultures of osteoblasts derived from knockout CD36 mice show reduced cell expansion and survival, as well as reduced expression of osteogenic-related genes and proteins [31]. Vascular endothelial cells play a key role in linking lipid metabolism and bone homeostasis. H-type vessels located in the metaphysis and under the periosteum, in addition to transporting lipids and metabolic products through endothelial cells to bone cells, may also produce intermediary signaling molecules that affect the energy metabolism of bone cells. This coupling between vessels, endothelial cells, and the bone metabolic microenvironment provides a theoretical basis for improving bone homeostasis by regulating the lipid metabolism of endothelial cells [22]. Based on this evidence, in recent years, numerous foundational studies and preclinical studies have focused on exploring the connections among statins, angiogenic drugs, and bone metabolism [32].

Initially, free fatty acids in the cellular matrix are activated to form fatty acyl-CoA. Subsequently, fatty acyl-CoA is converted to acyl-carnitine derivatives and transported into the mitochondrial matrix by carnitine palmitoyl transferase 1 (CPT1) and carnitine-acylcarnitine translocase (CAT). Within the inner mitochondrial membrane, fatty acyl-CoA is regenerated through the removal of carnitine by carnitine-palmitoyl transferase 2 (CPT2). Then, fatty acyl CoA undergoes β-oxidation to generate acetyl-CoA, which enters the tricarboxylic acid cycle and produces adenosine triphosphate (ATP) via oxidative phosphorylation. CAT is the key rate-limiting enzyme for acyl-CoA entry into mitochondria. Several studies have shown that carnitine supplementation promotes β-oxidation of fatty acids, stimulates the activity of osteoblasts and their progenitor cells, and promotes osteogenic differentiation; these processes improve ovariectomy-induced osteoporosis in animal models and promote fracture healing [33,34]. When CPT2 was specifically disrupted in osteoblasts and bone cells, CPT2 mutant mice exhibited notable deficiencies in postnatal bone formation [35] (Fig. 1).

The molecular signaling mechanisms regulating fatty acid β-oxidation in bone cells are complex. During the differentiation and maturation of osteoblasts and osteoclasts, as well as during different periods when osteoblasts perform mineralization functions and osteoclasts perform resorption functions, the cellular energy demands vary, leading to differences in the uptake and utilization of fatty acids [36]. Frey et al. [37] reported that the Wnt-Lrp5 signaling pathway plays a crucial role in regulating fatty acid metabolism in osteoblasts. Specifically, mice lacking Lrp5 in osteoblasts exhibit obesity, low bone mass, and impaired fatty acid β-oxidation.

Based on current research, several questions remain regarding the effect of fatty acids on the energy metabolism of skeletal cells. First, most current studies on energy metabolism in the skeleton have focused on fatty acid metabolism in the osteoblast lineage, with few studies on energy metabolism in the osteoblast lineage. As mentioned, the progenitor cells of osteoblasts, macrophages in the bone marrow microenvironment, contain fat droplets, but how these lipid droplets and foreign fatty acids generate a response according to the energy requirements of osteoblast differentiation and maturation is still poorly understood. Second, most existing studies on the effects of lipid energy metabolism on osteoblasts have focused on whether osteogenic differentiation is promoted or inhibited. The osteoblast lineage includes multiple cell types, ranging from BMSCs and pre-osteoblasts in the bone marrow niche to mature osteoblasts in the endosteum. Energy requirements may not be consistent at each developmental stage [38]; thus, further studies are needed to elucidate when and how fatty acids affect osteoblast differentiation and mineralization.

#### Fatty acid-related signaling pathways and bone homeostasis

Fatty acids act as signaling molecules, exerting their biological effects through interactions with receptors in skeletal cells. Specifically, cell membrane receptors [including GPRs and Toll-like receptors (TLRs)] and nuclear receptors [mainly peroxisome proliferator-activated receptors (PPARs)] of osteoblasts and osteoclasts, upon binding with fatty acids, induce transmembrane or intracellular signaling, leading to changes in the transcription and translation of target genes, thereby regulating cell differentiation, growth, behavior, and function. Considering the complexity of fatty acid classification, this section will discuss the effects of long-chain unsaturated fatty acids (LCUFAs), saturated fatty acids (SFAs), short-chain fatty acids (SCFAs), and medium-chain fatty acids (MCFAs) on bone cell metabolism. Based on the categories and names of fatty acids, potential receptors and signaling pathways, and their effects on specific bone cells, along with the corresponding references, are summarized in Table. The fatty acid signaling pathways and their effects on osteoblasts (including BMSCs), osteoclasts [including bone marrow-derived macrophages (BMMs)], and osteocytes, which are closely associated with bone homeostasis, are discussed.

**Table.** The signaling effects of fatty acids on specific cell types and their impact on bone homeostasis

Class	Fatty acid	Research type	Targeted cell	Receptor	Signaling pathway	Effect/conclusion	Year	References
ω-3 LCPUFAs	DHA	Vitro	BMSC	GPR 120	Akt/NF-κB	Osteogenesis ↑	2017	[26]
ω-3 LCPUFAs	DHA	Vitro	Osteoclast	NM*	c-Fos/NFATc1	Osteoclastogenesis↓	2015	[290]
ω-3 LCPUFAs	EPA/DHA	Vivo	Osteoblast	PPARγ	NF-κB	Osteogenesis↑ BMD↑	2021	[41]
			Osteoclast	GPR 40		Osteoclastogenesis↓		
ω-3 LCPUFAs	-	Vitro	Osteoclast	GPR 120	NM*	Uncertain outcome	2008	[291]
ω-3 LCPUFAs	DHA	Vitro	Osteoclast	GPR 120	NF-κB	Osteoclastogenesis↓	2010, 2014	[292,293]
ω-3 LCPUFAs	EPA	Vitro	Osteoclast	NM*	NM*	Osteoclastogenesis↑	2010	[292]
ω-3 LCPUFAs	EPA	Vitro	BMSC	GPR 120	PI3K/Akt	BMSC apoptosis↓	2016	[48]
ω-3 LCPUFAs	EPA/DHA	Vivo	Osteoclast	GPR 120	NF-κB	Osteoclastogenesis↓	2003	[294]
ω-3 LCPUFAs	EPA/DHA	Vivo	Osteoblast/Osteoblast	GPR 120/GPR 40	NF-κB	BMD↑ bone quality↑	2011	[295]
ω-3 LCPUFAs	DHA	Vitro/vivo	Osteoblast	GPR 120	Wnt/β-catenin	Osteogenesis ↑	2016	[296]
			Osteoclast			Osteoclastogenesis↓		
ω-3 LCPUFAs	DHA/EPA	Vitro/vivo	Osteoblast	GPR 120/GPR 40/	NF-κB	Osteogenesis↑ BMD↑	2021	[297]
			Osteoclast	GPR 120/GPR 40		Osteoclastogenesis↓		
ω-3 LCPUFAs	DHA	Vitro/vivo	Osteoclast	GPR 120	NF-κB	Osteoclastogenesis↓ BMD↑	2019	[60]
ω-3 LCPUFAs	DHA	Vitro	Osteoclast	GPR 120/GPR 40	NF-κB	Osteoclastogenesis↓ Osteoclasts apoptosis↑	2017	[298]
ω-3 LCPUFAs	DHA/EPA	Vitro	Osteoclast	GPR 120	NF-κB	Osteoclastogenesis↓	2019	[46]
			Osteoblast			Osteogenesis↑↑		
ω-3 LCPUFAs	DHA	Vitro	Osteoclast	GPR 120	NF-κB	Osteoclastogenesis↓	2023	[43]
ω-6 LCPUFAs	AA	Vitro	Osteoclast	NM*	NM*	Osteoclastogenesis↓	2015	[290]
ω-6 LCPUFAs	-	Vitro	Osteoclast	GPR 120/GPR 40	NM*	Uncertain outcome/Osteoclastogenesis↑	2008	[291]
ω-6 LCPUFAs	AA	Vitro	Osteoclast	NM*	NM*	Osteoclastogenesis↑	2010	[292]
ω-6 LCPUFAs	AA	Vitro	Osteoclast	NM*	NM*	Osteoclastogenesis↓	2014, 2018	[46,293]
ω-6 LCPUFAs	AA	Vitro	BMSC	NM*	NM*	Osteogenesis↓	2013	[51]
ω-6 LCPUFAs	AA	Vitro	BMSC	GPR 120/PPARγ2	NF-κB	Osteogenesis↓ adipogenesis↑	2013	[299]
Class	Fatty acid	Research type	Targeted cell	Receptor	Signaling pathway	Effect/conclusion	Year	References
LCMUFAs	Palmitoleic Acid	Vitro	Osteoblast/Osteoclast	GPR 120	NF-κB	Osteogenesis↑ Osteoclastogenesis↓	2019	[46]
LCMUFAs	Palmitoleic Acid	Vitro	Osteoclast	GPR 120/GPR 40	NF-κB	Osteoclastogenesis↓	2017	[47]
LCMUFAs	OA	Vitro/vivo	BMSC	TLR4	NF-κB	Osteogenesis ↑	2017	[300]
LCMUFAs	OA	Vitro	Osteoclast	NM*	NM*	Osteoclastogenesis↓	2014	[65]
SFAs	PA	Vivo	Osteoblast	NM*	NM*	Osteogenesis↓ BMD↓	2016	[301]
SFAs	PA	Vitro	Osteoblast	TLR4	MAPK/NF-κB	Osteoblast apoptosis↑ BMD↓	2022	[53]
SFAs	PA	Vitro	Osteocyte	NM*	NM*	Osteocyte apoptosis↑	2019	[55]
SFAs	PA	Vitro	Osteoclast	TLR4	NF-κB	Osteoclastogenesis↑	2014	[65]
SFAs	PA	Vivo	Osteoblast/Osteoclast	GPR40	NF-κB	BMD↑	2017	[67]
SFAs	PA	Vivo	Osteoclast	TLR2/TLR4	NF-κB	Osteoclastogenesis↑	2012	[77]
SFAs	Myristic oleic acid	Vitro	Osteoclast	GPR 40	NF-κB	Osteoclastogenesis↓	2015	[302]
SFAs	PA	Vitro	Osteoclast	TLR4	NF-κB	Osteoclastogenesis↑ osteoclast survival↑	2012	[66]
SCFAs	Acetate	Vitro	Osteoblast	GPR43	NF-κB	Osteogenesis↑	2022	[58]
	Propionate							
SCFAs	Butyrate	Vitro	Osteoclast	GPR43	NF-κB	Osteoclastogenesis↓	2021	[59]
	Acetate/Propionate		BMSC/Osteoblast			Osteogenesis↑		
SCFAs	Propionate	Vitro/vivo	Osteoclast	NM*	NM*	Osteoclastogenesis↓	2018	[71]
	Butyrate		BMSC/Osteoblast			Osteogenesis↑		
SCFAs	Propionate	Vitro/vivo	Osteoclast	-	NM*	Osteoclastogenesis↓	2022	[70]
	Butyrate			GPR109A/GPR41	NF-κB			
SCFAs	-	Vitro/vivo	Osteoclast	GPR43	NF-κB	Osteoclastogenesis↓	2019	[72]
						Osteogenesis↑		
MCFAs	Caprylic acid	Vitro/vivo	BMSC	PPARγ	PI3K-Akt	Osteogenesis↓ adipogenesis↑	2020	[56]
MCFAs	Octanoic acid	Vivo	Osteoblast/Osteoclast	NM*	NM*	BMD↓	2021	[57]
MCFAs	Capric acid	Vitro	Osteoclast	NM*	STAT3	Osteoclastogenesis↓	2011	[303]
MCFAs	Capric acid	Vitro	Osteoclast	GPR	NF-κB	Osteoclastogenesis↓ Osteoclast apoptosis↑	2014	[68]

NM*, not mentioned

**Fig. 2.**
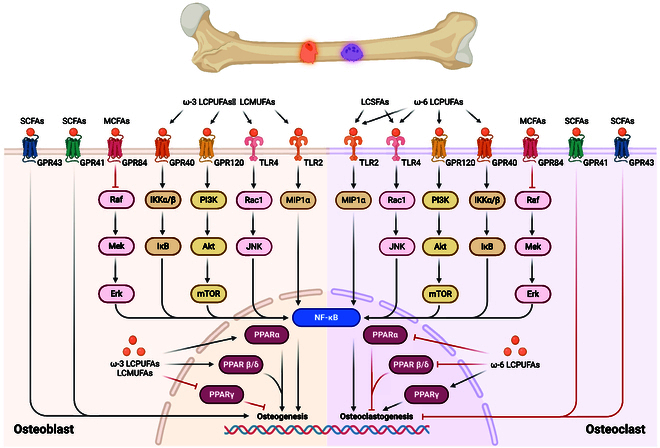
Fatty acid-related signaling pathways. Signaling pathways of fatty acids affecting the activity and function of osteoblasts and osteoclasts.

Osteoblasts: Fatty acids, as signaling molecules, significantly influence BMSCs by interacting with specific receptors. LCUFAs, including ω-3 and ω-6 polyunsaturated fatty acids (LCPUFAs), as well as long-chain monounsaturated fatty acids (LCMUFAs), play crucial roles in regulating BMSC differentiation. ω-3 LCPUFAs are known for their anti-inflammatory [39] and osteogenic effects [40], as they promote bone formation and improve bone density by up-regulating the osteogenic differentiation of BMSCs [26,41–43]. ω-6 LCPUFAs often exhibit proinflammatory properties, leading to reduced osteogenic differentiation and increased bone resorption [44]. The signaling mechanisms involved in ω-3 LCPUFA-mediated osteoblast differentiation include the activation of the Wnt/β-catenin pathway, which is crucial for promoting osteogenesis. Studies have demonstrated that supplementation with ω-3 LCPUFAs in obese osteoporotic mice significantly up-regulated the expression of osteogenic markers, leading to increased bone formation and improved bone mineral density (BMD) [45]. Conversely, ω-6 LCPUFAs promote the synthesis of arachidonic acid (AA) and proinflammatory eicosanoids such as prostaglandins, which can inhibit osteoblast differentiation and function [39]. In addition, LCMUFAs, such as ω-5, ω-7, and ω-9 LCMUFAs, are commonly considered potential agents against disorders of bone metabolism; more specifically, they promote osteogenesis, inhibit osteoclastogenesis, and ultimately facilitate bone formation [46,47]. LCPUFAs exert their effects primarily through GPR40 and GPR120 receptors (Table and Fig. 2). For example, eicosatetraenoic acid (EPA), an ω-3 LCPUFA, can induce adaptive autophagy in BMSCs by binding to GPR120, counteracting apoptosis, and facilitating differentiation into osteoblasts [45,48]. Additionally, in the nucleus, ω-3 LCPUFAs have been shown to act as ligands for PPARα and PPARβ/δ, promoting osteogenesis [49], while ω-6 LCPUFAs activate PPARγ, which shifts BMSC differentiation toward adipogenesis rather than osteogenesis [50,51]. This differentiation shift is crucial because it highlights the balance between ω-3 and ω-6 fatty acids maintained within BMSCs, influencing whether these stem cells become bone-forming cells or fat-storing cells.

Moreover, SFAs, such as PA, can have adverse effects on osteoblasts (Table). For instance, PA induces lipotoxicity, reducing osteogenic differentiation by decreasing alkaline phosphatase (ALP) activity and the expression of key osteogenic genes such as RUNX2 and osterix [52]. The activation of TLR4 by SFAs in osteoblasts leads to increased endoplasmic reticulum stress and inflammatory responses, which inhibit osteoblast differentiation and mineralization. Studies have shown that PA can induce apoptosis in mature osteoblasts [53], further contributing to decreased bone formation and increased bone resorption. However, it has also been observed that the negative effects of PA on osteoblasts can be mitigated by the induction of autophagy, which helps to restore the osteogenic potential of these cells [54,55].

MCFAs contain 6 to 12 carbon atoms in their carbon chain and are primarily composed of caprylic acid and capric acid. In the osteoblast lineage, certain in vitro experiments have shown that treatment of BMSCs with octanoic acid significantly up-regulates lipogenic markers such as CCAAT/enhancer binding protein α and PPAR γ while concurrently suppressing osteogenic-related genes such as RUNX2 and OPG. This suggests that octanoic acid skews the regulation of the lipogenic–osteogenic balance in the bone marrow toward lipogenic differentiation, thereby inhibiting osteoblast differentiation [56]. Correspondingly, in vivo experiments have shown notable detrimental changes in trabecular bone microarchitecture in octanoic acid-treated mice, revealing significant reductions in BMD and bone mineral content, as well as diminished levels of ALP [57] (Table).

SCFAs, primarily acetate, propionate, and butyrate, are predominantly produced through the fermentation of dietary fiber by the intestinal microbiome, although they may also originate from skin and vaginal bacteria. Existing studies overwhelmingly suggest that SCFAs have a beneficial effect on bone homeostasis (Table). In vitro experiments showed that the action of acetate and propionate on primary osteoblasts significantly up-regulated ALP activity and promoted early osteoblast differentiation [58]. Wallimann et al. [59] further demonstrated that coculturing butyrate with BMSCs resulted in increased calcium deposition by BMSCs while concurrently reducing osteoclast formation and resorption activity in a dose-dependent manner. In vivo experiments showed that by supplementing mice with Lactobacillus rhamnosus GG, which produces butyrate in the intestine, or by feeding them directly with butyrate, microcomputed tomography showed an increase in trabecular bone volume in mice, suggesting that SCFAs have a positive effect on bone anabolism.

Osteoclasts: Osteoclasts, which are responsible for bone resorption, are significantly influenced by fatty acids through various signaling pathways. Numerous studies have reported that ω3-LCPUFAs directly inhibit the differentiation and activity of osteoclasts [60,61]. In contrast to the effects of ω-3 LCPUFAs, elevated levels or a high ratio of ω-6 LCPUFAs increases bone loss by promoting chronic inflammation, reducing BMSC osteogenic differentiation, activating osteoclasts, and promoting osteoclast maturation. ω-3 LCPUFAs inhibit osteoclast differentiation and activity mainly by binding to GPR120 and GPR40 [62] (Table and Fig. 2). However, the effects of LCUFAs on PPARs in the osteoclast lineage are currently controversial. Nakanishi and Tsukamoto [63] reported that ω-3 LCPUFAs enhance osteoclast differentiation in BMMs by activating PPAR-γ; nevertheless, simultaneous activation of PPAR-γ in MSCs inhibits NF-κB activation to suppress osteoclast formation. Recently, Kasonga et al. [64] showed that the activation of all PPARs by LCUFAs down-regulates the activation of the RANKL signaling pathway and inhibits osteoclast formation.

SFAs promote osteoclast activity and bone resorption. PA, for example, enhances osteoclastogenesis by activating TLR4, leading to increased NF-κB signaling and osteoclast survival (Table and Fig. 2). The proinflammatory environment created by SFAs stimulates osteoclast differentiation and function, resulting in increased bone resorption and decreased bone density [65]. SFAs can induce the production of macrophage inflammatory protein-1α (MIP-1α) and promote the survival of mature osteoclasts, further exacerbating bone loss [66]. In contrast, the effects of SFAs on the activation of GPRs may be controversial. Philippe et al. [67] reported that when C57/BL6 female mice were stimulated with a diet rich in SFAs, the activation of GPR40 regulates the OPG/RANKL/NF-κB axis, reducing NF-κB activation and decreasing osteoclastogenesis, which helps to counteract ovariectomy-induced bone loss; however, this in vivo experiment failed to exclude the interfering effects of other fatty acids.

MCFAs tend to impede osteoclast formation. In vitro studies revealed that decanoic acid diminishes the viability and differentiation of osteoclasts by suppressing NF-κB signaling while enhancing the apoptosis of mature osteoclasts through the inhibition of extracellular signal-related kinase (ERK) activation [68]. Similarly, in vivo experiments involving mice fed caprylic acid for 4 weeks revealed an increase in the specific marker tartrate-resistant acid phosphatase (TRAP) of osteoclasts. Therefore, based on existing evidence, MCFAs appear to exert inhibitory effects on the differentiation, maturation, and function of osteoclasts. MCFAs exert their signaling effects by binding to the GPR84 receptor on the cell membranes of bone cells (Table and Fig. 2). Activation of GPR84 leads to down-regulation of the ERK/NF-κB signaling pathway, thereby reducing the differentiation and maturation of osteoblasts and osteoclasts [56,68,69]. Notably, in addition to the GPR84 receptor, other receptor signaling systems may be involved, and the existing ERK/NF-κB signaling pathway may interact with other signaling pathways. Addressing these questions may necessitate further in-depth basic research.

In osteoclasts, SCFAs inhibit osteoclastogenesis and down-regulate osteoclastic resorptive function [59]. Notably, propionate and butyrate effectively inhibit CoCrMo alloy particle-induced macrophage pyroptosis and osteoclastogenesis, thereby mitigating wear particle-induced osteolysis [70]. These SCFAs induce metabolic reprogramming of osteoclasts, enhancing glycolysis at the expense of oxidative phosphorylation and down-regulating essential osteoclast genes such as tumor necrosis factor receptor-associated factor 6 (TRAF6) and nuclear factor of activated T cells cytoplasmic 1 (NFATc1) [71]. The main receptors for SCFAs in skeletal cells include GPR43 and GPR41, which are located on the cell membrane (Table and Fig. 2). Bone samples from GPR43-deficient mice (GPR43^−/−^) receiving a high-SCFA diet reportedly show increased osteoclast differentiation and activity, while bone markers from wild-type mice favor bone formation [72]. These results suggest that SCFAs promote osteoblast differentiation and limiting osteoclast activation in response to GPR43 activation.

Osteocytes: Osteocytes, the most abundant cells in bone, play a critical role in maintaining bone homeostasis by regulating the activity of osteoblasts and osteoclasts. Emerging evidence suggests that fatty acids, integral components of cellular membranes and precursors to signaling molecules, significantly influence osteocyte function. ω-3 LCPUFAs, particularly EPA and DHA, are known for their anti-inflammatory properties [73]. They influence osteocyte signaling by modulating the production of pro-inflammatory cytokines and eicosanoids. EPA and DHA can be converted into specialized pro-resolving mediators such as resolvins and protectins, which reduce inflammation and oxidative stress in osteocytes [39]. This modulation occurs through the activation of G GPRs like GPR120, which subsequently triggers intracellular signaling cascades that enhance osteocyte survival and function. ω-3 LCPUFAs also impact the Wnt/β-catenin pathway, a crucial signaling route for bone formation and osteocyte function. By enhancing this pathway, EPA and DHA promote the survival and activity of osteocytes [74]. While ω-6 LCPUFAs such as AA are necessary for normal cellular functions, their metabolites (prostaglandins and leukotrienes) can promote inflammation. The balance between ω-3 LCPUFAs and ω-3 LCPUFAs is crucial in osteocytes. High levels of AA-derived metabolites can exacerbate inflammation, potentially leading to osteocyte apoptosis and impaired bone remodeling [45]. Conversely, controlled levels of AA can support osteocyte function by participating in the synthesis of signaling molecules that are essential for cellular communication and bone homeostasis [75].

SFAs, typically found in animal fats and some plant oils, have a distinct influence on cellular signaling due to their structural characteristics. SFAs contribute to the rigidity of cell membranes, impacting the function of membrane-bound proteins and receptors involved in signaling transduction [76]. Excessive SFA intake can lead to altered membrane properties, impairing the fluidity required for optimal receptor function and signaling molecule diffusion. High levels of SFAs are associated with increased production of pro-inflammatory cytokines through the activation of TLRs, particularly TLR4 [77]. This activation can induce chronic inflammation in osteocytes, leading to apoptosis and disrupted bone homeostasis. SFAs may also influence osteocyte signaling by affecting the NF-κB pathway [78], a key regulator of inflammation and cell survival. Chronic activation of NF-κB can detrimentally affect osteocyte viability and function.

MCFAs possess unique metabolic properties that differentiate them from long-chain fatty acids. Unlike SFAs, MCFAs have been shown to have a neutral or even beneficial effect on inflammation, potentially by modulating mitochondrial function and reducing oxidative stress [79]. MCFAs may influence osteocyte signaling by modulating the PPAR pathway. Activation of PPARs, particularly PPARδ, supports lipid metabolism and energy homeostasis in osteocytes [80], promoting their survival and function. MCFAs also affect the adenosine 5-monophosphate activated protein kinase (AMPK) pathway, a critical regulator of cellular energy status. Activation of AMPK enhances osteocyte autophagy and stress response mechanisms, contributing to improved cell survival under metabolic stress [81].

The SCFAs have systemic effects beyond the gut, influencing bone metabolism and osteocyte function. SCFAs influence osteocyte signaling through the gut–bone axis [82]. They are absorbed into the bloodstream and can affect bone cells directly. SCFAs, particularly butyrate [83], have been shown to enhance osteocyte viability and function by modulating histone deacetylase (HDAC) activity [84]. Inhibition of HDAC by butyrate promotes the expression of genes involved in osteocyte survival. SCFAs also influence the production of gut-derived hormones such as glucagon-like peptide-1, which have been shown to impact bone metabolism and osteocyte function indirectly.

In summary, fatty acids play a significant role in regulating the differentiation and function of bone cells through interactions with specific receptors and signaling pathways. LCUFAs, particularly ω-3 and ω-6 LCPUFAs, have differential effects on bone metabolism, with ω-3 LCPUFAs generally promoting osteogenesis and anti-inflammatory responses, while ω-6 LCPUFAs exhibit proinflammatory properties and inhibit osteogenesis. SFAs generally have detrimental effects on bone cells, promoting inflammation and bone resorption, while MCFAs and SCFAs exhibit beneficial effects on bone health by inhibiting osteoclastogenesis and promoting osteogenesis. Understanding the precise mechanisms by which these fatty acids exert their effects on bone cells is crucial for developing dietary and therapeutic strategies to enhance bone health and prevent bone-related diseases. Future research should focus on elucidating the specific signaling pathways and receptors involved in fatty acid regulation of bone metabolism to provide a comprehensive understanding of the interplay between dietary fatty acids and bone health.

#### Clinical studies and applications

In recent years, a large number of clinical observational studies have described the therapeutic potential of fatty acids, especially long-chain fatty acids, in bone metabolic diseases. Most of the current epidemiological surveys and human studies have shown that increasing the dietary intake of ω-3 LCPUFAs or the ratio of ω-3 LCPUFAs to ω-6 LCPUFAs improves human bone structure [85–87]. In addition, ω-3 LCPUFAs may have another beneficial effect on bone homeostasis by mitigating the side effects of bone-targeted drugs. Fan et al. [88] reported that the intake of ω-3 LCPUFAs alleviates bone marrow injury and bone tissue toxicity induced by azacitidine or cyclophosphamide chemotherapy in rats.

The main cause of osteoporosis is an imbalance between overactive osteoclasts and inactive osteoblasts, leading to excessive bone resorption. Animal studies have shown that ω-3 LCPUFAs attenuate osteoporosis by inhibiting bone destruction, increasing dietary calcium absorption, and increasing skeletal BMD [89]. Mechanistically, ω-3 LCPUFAs inhibit osteoclastogenesis and decrease low-grade chronic inflammation, thereby contributing to the prevention and alleviation of osteoporosis. Furthermore, the intake of ω-6 LCPUFAs leads to an increase in the ratio of ω-6 to ω-3 LCPUFAs, which promotes osteoporosis by facilitating low-grade inflammation and suppressing the osteogenesis of MSC lineages [90]. Studies have also revealed that because of the susceptibility of LCPUFAs to reactive oxygen species (ROS)-associated oxidative damage, treatment with the antioxidant CoQ during osteoporosis treatment could reduce the drawbacks of LCPUFAs [91]. However, the correlation between fatty acids and osteoporosis has not been consistent in studies conducted in humans.

Bone fracture is the most serious complication of osteoporosis. Chen et al. [92] showed that supplementation with ω-3 LCPUFAs promoted fracture healing in a mouse fracture model. In addition, several clinical studies have shown that increasing total PUFA intake is beneficial for maintaining BMD and reducing fracture risk [87]. However, an epidemiological survey showed that reduced intake of total PUFAs and ω-6 LCPUFAs increased the risk of hip fracture in women [93]. This discrepancy may be due to differences in data processing, sample inclusion, and study design. Moreover, the diversity of fatty acid types may be another important factor. In conclusion, different types of fatty acids may affect fractures differently. Thus, additional investigations are needed to elucidate the specific underlying mechanisms involved.

Rheumatoid arthritis is a chronic inflammatory disease associated with autoimmune abnormalities that usually leads to cartilage and bone erosion, causing primary joint destruction. Studies have shown that ω-3 LCPUFAs attenuate the symptoms of rheumatoid arthritis, including joint swelling and morning stiffness, by inhibiting inflammatory cytokines and cartilage-degrading enzymes [94]. In addition, the LCPUFA AA promotes the synthesis of proinflammatory cytokines. Therefore, concomitant restriction of the LCPUFA AA may enhance the anti-rheumatoid arthritis effects of ω-3 LCPUFAs by reducing the production of metalloproteinases and proinflammatory cytokines [94]. ω-6 LCPUFAs, another type of LCPUFA, are ultimately metabolized to AA and exert proinflammatory effects. In contrast, ω-3 LCPUFAs interact with the delta-6 desaturase enzyme, which is the rate-limiting synthase of ω-6 LCPUFAs, resulting in a therapeutic effect on rheumatoid arthritis [95].

It should be noted that the data from current observational studies are highly heterogeneous, and the evidence level is not high; therefore, the conclusions of these studies need to be treated with caution. In summary, fatty acids may have significant potential in improving bone homeostasis and treating bone metabolic diseases. Considering that our daily diet contains a substantial quantity of fatty acids, further research into the effects of fatty acids on bone metabolism and their potential mechanisms would be valuable for exploring their beneficial effects on various metabolic bone diseases. If possible, using “food therapy” instead of “drug therapy” to treat diseases like osteoporosis would be highly valuable.

### Cholesterol participates in bone homeostasis

#### The sources and metabolic pathways of cholesterol in bone homeostasis

The sources of cholesterol in bone cells include exogenous uptake and endogenous synthesis. Exogenous cholesterol intake mainly involves the processing of dietary cholesterol by digestive enzymes, which are initially packaged into chylomicrons and enter the blood circulation [96]. Through hepatic reassembly, most chylomicrons circulate in the blood as LDL, which is then captured by LDLR on the surface of target cells. This leads to LDL-LDLR endocytosis and further intracellular hydrolysis of LDL, eventually producing free cholesterol [97]. Endogenous cholesterol synthesis involves the following main steps: First, 2 acetyl-CoA molecules are reverse-catalyzed by thiolate to produce acetoacetic-CoA; subsequently, 3-hydroxy-3-methylglutaryl coenzyme A (HMG-CoA) is generated via the catalysis of HMG-CoA synthase using acetoacetic-CoA and acetyl-CoA, which is then converted to mevalonate via a rate-limiting step catalyzed by HMG-CoA reductase (HMGCR). Mevalonate undergoes a series of phosphorylation and decarboxylation reactions to form isopentenyl pyrophosphate, which is then converted to dimethylallyl pyrophosphate. These 2 compounds are condensed to produce geranyl pyrophosphate, which undergoes further condensation with isopentenyl pyrophosphate to form farnesyl pyrophosphate. Squalene synthase catalyzes the condensation of 2 molecules of farnesyl pyrophosphate to form squalene. Finally, squalene undergoes cyclization, facilitated by specific cyclase enzymes, to produce lanosterol, which is subsequently converted into cholesterol [98,99]. Given its pivotal role in cholesterol synthesis, HMGCR serves as a crucial target for drug intervention aimed at regulating cholesterol levels. Modulating HMGCR activity can significantly impact managing cholesterol-related disorders and may offer therapeutic benefits in various clinical settings (Fig. 1).

In a healthy state, cholesterol levels in the body are regulated through a complex interplay of factors, including dietary intake, endogenous synthesis, transport, cellular uptake, and excretion. This intricate balance ensures cholesterol homeostasis. However, disturbances in this equilibrium, such as hypercholesterolemia, often result from a combination of environmental factors, such as obesity, unhealthy diet, and stress, along with genetic predisposition [100]. While there is a direct relationship between dietary cholesterol intake and serum cholesterol levels in some cases, the circulating levels of cholesterol are also influenced by other dietary components. For instance, the intake of SFAs, trans fatty acids, dietary soluble fiber, and fructose can significantly impact cholesterol metabolism. Consequently, maintaining normal cholesterol levels is vital for overall health. However, excessive cholesterol accumulation can have detrimental effects on cellular function and is implicated in the dysregulation of bone homeostasis.

#### Cholesterol and cellular membranes in bone tissue

**Fig. 3.**
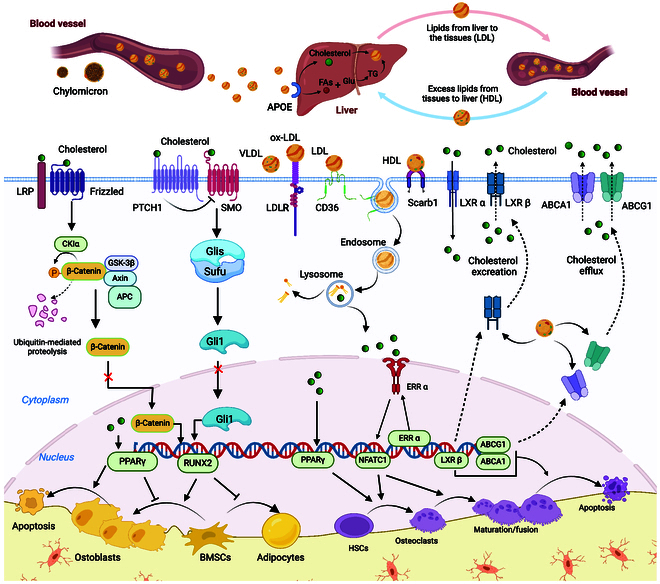
Regulation of bone homeostasis by cholesterol. (1) Exogenous cholesterol inhibits the osteogenic differentiation of BMSCs by down-regulating the typical Wnt/β-catenin signaling pathway and hedgehog signaling pathway. (2) Lipoprotein particles enter the cytoplasm by “internalization” and are hydrolyzed by lysosomal acid lipase to release free cholesterol. (3) Cholesterol increases osteoclast differentiation while promoting osteoclast maturation and fusion by activating ERRα and PPARγ in the nucleus. (4) LXR, ABCA1, and ABCG1 regulate the inflow and outflow of cholesterol and HDL retrograde cholesterol transport, which together maintain physiologic intracellular cholesterol concentrations.

Cell membranes are vital barriers for cells and serve as the primary interface for the extracellular environment. Among the essential components of the plasma membrane, cholesterol is particularly important for its influence on the physical characteristics of the lipid bilayer. Predominantly hydrophobic, like other steroids, cholesterol is synthesized by all mammalian cells and is mainly situated within the cell membrane [101]. Cholesterol in the lipid bilayer regulates its rigidity, fluidity, and permeability, thereby influencing the overall membrane structure. Additionally, cholesterol interacts with various transmembrane proteins, modulating their conformation and function. Cholesterol also associates with sterol transport proteins, facilitating their intracellular movement and distribution. Furthermore, cholesterol collaborates with lipid–protein complexes such as lipid rafts, contributing to the organization of signaling molecules within membrane microdomains. This interplay potentially impacts the regulation of stem cell behavior and stem cell responsiveness to external stimuli. Thus, cholesterol plays a multifaceted role in shaping cell membrane dynamics and cellular responses [102] (Fig. 3).

Disturbances in cholesterol, caveolin-1, and caveolar homeostasis are crucial for regulating the membrane properties and adhesion characteristics of BMSCs. Supplementing cholesterol in BMSCs enhances membrane cholesterol levels, reduces membrane fluidity, and promotes the accumulation of caveolae and caveolin-1 on the cell membrane. An increase in the cellular membrane cholesterol content may increase the sensitivity of BMSCs to osteogenic induction cues [103–105]. Studies have shown that statins, which lower endogenous cholesterol, impair the osteogenic potential of BMSCs and increase cellular senescence and apoptosis [106]. Although these studies do not explicitly identify the specific mechanisms by which statin drugs impair stem cell osteogenic differentiation, it can be inferred that depletion of cellular membrane cholesterol may have a negative effect on the behavior and activity of osteogenic cells. Similarly, the membrane homeostasis of cholesterol in osteoclast cell lines plays an important role in the formation, survival, differentiation, and activity of osteoclasts [107]. Recent findings have established that the disruption of cholesterol homeostasis in osteoclast membranes compromises lipid raft functionality, inhibits osteoclast formation, and triggers osteoclast apoptosis [108] (Fig. 3).

However, the role of cholesterol in cellular biofilms and its impact on bone homeostasis have been marginally explored, suggesting that we may only be seeing the beginning of its implications. The complexity of biofilms is a major obstacle to further understanding; this complexity includes both chemical diversity (the variety of lipid structures within cells) and compositional diversity (the proportions of different lipids and proteins). Chemical diversity imparts unique characteristics to lipids, while compositional diversity affects the overall behavior of lipids in biofilms. Furthermore, it often remains uncertain how variations in lipid composition or structural abnormalities affect disease and phenotypes. Even slight modifications in the cell membrane structure can interfere with the entire metabolic pathway, presenting a challenge in studying the effects of cholesterol.

#### Cholesterol-related signaling pathways and bone homeostasis

Cholesterol might act as a signaling molecule that regulates bone cells. In osteoblasts and osteoclasts, cholesterol signaling at different concentrations may initiate different signaling pathways and produce different effects. H-type vessels also play a crucial role in linking cholesterol and the metabolism of bone cells. Cholesterol and various lipoproteins are initially engaged through scavenger receptor B1 (SR-B1) or activin A receptor-like kinase 1 on the apical side of endothelial cells, initiating cellular endocytosis [109]. Through processing by endothelial cells, cholesterol or its metabolic products establish signaling connections with osteoblasts, osteoclasts, or osteocytes.

Osteoblasts: Numerous cellular and animal models have been used to investigate the impact of both serum cholesterol and cellular cholesterol on the proliferation and differentiation of osteoblasts. You et al. [110] reported that in vitro, the proliferation and viability of osteoblasts were inhibited by free cholesterol in a concentration-dependent manner. A recent high-quality literature suggests that cholesterol levels in osteoblasts affect the process of bone formation. Excessive free cholesterol inhibits the expression of key bone formation factors such as BMP2, which reduces the expression of RUNX2, ALP, and Col I a1 and inhibits osteoblast differentiation, while lecithin-cholesterol acyltransferase (LCAT) promotes reverse cholesterol transport, which prevents cholesterol from accumulating in excess in osteoblasts, thereby improving osteoblast survival [111].

It has been shown that exogenous cholesterol can modulate the activity of 7 transmembrane receptor binding sites on cell membranes and can down-regulate Wnt/β-catenin signaling or hedgehog signaling [112]. Cholesterol transmits the hedgehog signal from the transporter-like receptor Patched1 (PTCH1) to Smoothened (SMO) [113] (Fig. 3). During bone development, hedgehog signaling controls the differentiation of BMSCs into chondrocytes and osteoblasts during endochondral ossification by increasing the protein expression of RUNX2 and its downstream target osterix. Inhibition of SMO leads to down-regulation of the hedgehog signaling pathway [114]. Similarly, Li et al. [115] reported that exogenous cholesterol significantly inhibited hedgehog signaling, as evidenced by a decrease in Gli1, a marker of hedgehog signaling, and a decrease in osteogenic markers during the osteogenic differentiation of BMSCs. However, depletion of endogenous cholesterol, which affects only the hedgehog signaling pathway, also reduced the osteogenic response, suggesting the existence of a separate mechanism by which cholesterol regulates osteogenic differentiation independent of hedgehog signaling. Indeed, it has been documented that exogenous cholesterol is directly involved in the regulation of the typical Wnt/β-catenin signaling pathway [116] and that down-regulation of the Wnt/β-catenin signaling pathway may inhibit osteogenic differentiation in BMSCs [117].

As carriers of circulating cholesterol, lipoproteins are essential for the maintenance of cellular cholesterol homeostasis. LDLR, scarb1, and CD36 mediate the endocytosis of lipoprotein particles such as LDL, VLDL, oxidized LDL (ox-LDL), and others (Fig. 3), and intracellularly, these endosomes fuse with lysosomes and are hydrolyzed by lysosomal acid lipase to release free cholesterol [118]. Physiological concentrations of intracellular cholesterol are essential for maintaining cellular activity and differentiation properties [32]. Under pathological conditions such as hypercholesterolemia, LDL, VLDL, and ox-LDL are generally detrimental factors for the differentiation and function of osteoblasts. In vitro investigations demonstrated that overloading ox-LDL suppresses stromal cell osteoblastic differentiation by impeding ALP activity, Col I a1 processing, and mineralization [119].

Osteoclasts: Overall, cholesterol signaling has been shown to play a role in up-regulating osteoclast differentiation and maturation in osteoclast cell lines. Cholesterol alters the autophagic capacity of osteoclasts, increases their viability, and promotes osteoclast differentiation by activating the phosphatidylinositol 3-kinase (PI3K)/Akt/mammalian target of rapamycin (mTOR) signaling pathway [120]. Sul et al. [121] also reported that cholesterol increased the size, number, fusion index, and bone resorption activity of osteoclasts, indicating that high cholesterol levels increase the number and activity of osteoclasts. While in vivo experiments showed that a high-cholesterol diet increased the number and surface area of TRAP-stained osteoclasts, cholesterol depletion reduced osteoclast formation and bone resorption. This evidence suggests that cholesterol plays a critical role in inducing osteoclast activation and bone loss.

The effect of free cholesterol signaling on osteoclast biology is mediated mainly through liver X receptors (LXRs) localized to the cell membrane and estrogen-related receptor α (ERRα) localized to the nucleus. The expression of LXRs on the cell surface, including LXR and LXRβ, plays important roles in osteoclast differentiation and maintenance of osteoblast activity by regulating intracellular cholesterol homeostasis [122]. On the one hand, LXRα may mediate cholesterol uptake to maintain osteoclast activity, and mice in which LXRα was specifically knocked down showed a significant increase in BMD, and a large number of osteoclasts with lower activity than that of normal cells were detected in the cortical bone of the mice. On the other hand, the activation of LXRβ promotes the excretion of intracellular cholesterol and reduces the uptake of extracellular cholesterol, which is regarded as a limiting factor in osteoclast differentiation and maturation [123,124]. Lu et al. [111] showed that intracellular cholesterol levels affect osteoclast maturation and function. Removal of intracellular cholesterol prevents RANKL- or CSF-1-induced osteoclast formation, and LCAT reduces intracellular cholesterol levels by accelerating cholesterol efflux, thereby inhibiting osteoclast maturation and activity. This process may be achieved by disrupting the transcriptional activity of ERRα. In the cell nucleus, as an ERRα ligand, cholesterol enhances the transcriptional activity of osteoclast-specific genes such as NFATc1, resulting in further enhancement of osteoclast differentiation, while the use of β-cyclodextrin-threaded polytrioxanes to eliminate cholesterol overload reverses the increase in osteoclast differentiation [125,126].

Different types of apolipoproteins produce multiple biological effects on osteoclasts. While increasing LDL levels in conditioned media in vitro significantly up-regulates osteoclast viability [127,128]. Osteoclast formation is reduced when cells are cultured in LDL-depleted serum, and supplementation with ox-LDL reverses impaired osteoclastogenesis in LDL-depleted serum [129]. In contrast, HDL increases the efflux of intracellular cholesterol by increasing the expression of LXRβ, ABCA1, and ABCG1 at the cell membrane. Reverse cholesterol transport reduces the lipotoxicity caused by cholesterol overload on osteoblasts and simultaneously promotes the apoptosis of mature osteoclasts, which is thought to be beneficial for bone formation [108,130]. Scarb1 may play an important role in HDL internalization and cholesterol efflux. In vitro, knocking down Scarb1 in osteoblasts leads to reduced osteogenic differentiation; additionally, in vivo results demonstrated that mice lacking scarb1 exhibit a low bone mass phenotype characterized by decreased bone density and bone volume fraction [131]. All in all, lipoproteins affect the formation, survival, maturation, and apoptosis of osteoblasts and osteoclasts through the regulation of cholesterol homeostasis. This provides a theoretical basis and support for the medical regulation or improvement of lipid profiles to reduce bone loss and promote bone health.

Osteocytes: A plenty of recent research has highlighted the significance of cholesterol and lipoproteins in osteocyte signaling transduction, impacting various cellular functions. Cholesterol, as an essential lipid, is a critical component of cell membranes, influencing membrane fluidity and the function of membrane-bound receptors and ion channels. In osteocytes, cholesterol’s role extends to several key signaling pathways: First, cholesterol-rich microdomains, known as lipid rafts, serve as platforms for the assembly of signaling molecules. These rafts facilitate the clustering of receptors and downstream signaling proteins, enhancing signal transduction efficiency. For osteocytes, lipid rafts are vital for mechanical transduction—the process by which mechanical stimuli are converted into biochemical signals [132]. Cholesterol modulates the activity of ion channels and receptors involved in sensing mechanical stress, thus influencing bone remodeling and adaptation to mechanical load. Second, cholesterol interacts with components of this pathway, affecting the stability and activity of β-catenin, and studies suggest that cholesterol depletion impairs Wnt signaling, leading to reduced osteocyte activity and bone formation [133]. Conversely, adequate cholesterol levels support robust Wnt signaling, promoting osteocyte survival and function.

Lipoproteins, responsible for the transport of cholesterol and other lipids in the bloodstream, also play a significant role in osteocyte signaling: LDL is often referred to as “bad cholesterol” due to its association with cardiovascular disease. However, LDL and its receptor (LDLR) are important for cholesterol delivery to osteocytes [128]. Excessive LDL can undergo oxidation, forming ox-LDL, which is detrimental to osteocyte function [119]. Ox-LDL induces oxidative stress and inflammation, similar to oxysterols, leading to osteocyte apoptosis and impaired bone metabolism. HDL, known as “good cholesterol”, facilitates the reverse transport of cholesterol from tissues back to the liver for excretion. HDL exerts protective effects on osteocytes by reducing oxidative stress and inflammation. HDL and its associated enzymes, such as paraoxonase-1, help neutralize ox-LDL and other oxidized lipids, thus preserving osteocyte viability and function [131]. Enhanced HDL levels are associated with improved bone density and reduced risk of osteoporosis. Osteocytes express various lipoprotein receptors, including LDLR and scavenger receptors like scavenger receptor A (SR-A) and CD36. These receptors mediate the uptake of lipoproteins and their associated lipids. Through these receptors, lipoproteins influence intracellular signaling pathways, including those regulating lipid metabolism, oxidative stress, and inflammatory responses [134]. Proper function of these receptors is crucial for maintaining osteocyte health and bone homeostasis.

#### Clinical applications: Diseases and interventions

Over the past few decades, epidemiological data have provided compelling evidence linking serum cholesterol levels to bone health [135–140]. However, it is important to acknowledge that most of these investigations have significant limitations, including insufficient evidence from cross-sectional studies and even conflicting conclusions among different studies. These limitations may be attributed to the heterogeneity of clinical investigations themselves and inconsistencies in cholesterol and lipoprotein detection methods. Nevertheless, the presentation of this clinical evidence is important because it potentially suggests that maintaining an appropriate total cholesterol concentration may help prevent bone loss. Moreover, certain serum biomarkers, such as free cholesterol, LDL-C, and HDL-C, may serve as responsive indicators for the early detection of osteoporosis and guide treatment strategies in the future.

Cholesterol regulates bone metabolism in multiple ways, and vitamin D, an important metabolite of cholesterol, plays a crucial role in the maintenance of skeletal bone mass [141]. It is well known that elevated cholesterol promotes arterial calcification, and interestingly, high levels of cholesterol are negatively correlated with bone density [142]. A previous cross-sectional study in a Spanish population revealed a negative correlation between serum total cholesterol levels and 25(OH) vitamin D levels [143]. In addition, patients with familial hypercholesterolemia had reduced BMD [144]. Animal studies have shown that a high-cholesterol diet leads to osteoblast insufficiency and increased numbers of osteoclasts [141].

The association between serum cholesterol levels and skeletal health suggests the potential therapeutic value of cholesterol-lowering drugs in improving bone homeostasis. One notable example is the exploration of statins for reducing bone loss. Several preclinical studies have indicated that in osteoblast cell lines, statins increase osteoblast viability, osteogenic differentiation potential, and mineralization capacity [145–148]. In osteoclast cell lines, numerous studies have reported the inhibitory effects of statins on osteoclast differentiation and activity, leading to a reduction in bone loss and potential therapeutic benefits in osteoporosis [149–155]. Consistent with the findings of the preclinical studies described above, most of the observational clinical studies have confirmed the effectiveness of statins in improving BMD and reducing fracture risk [156–159]. It is important to recognize that the results derived from these observational and secondary studies are not conclusive. Considering the heterogeneity of observational data on the effects of statin therapy on bone formation markers, BMD, and fracture risk, as well as the dynamic changes in human lipids and BMD, it is challenging to obtain and analyze these data in a clinical setting [151]. Coupled with the presence of many confounding factors in observational studies and differences in patient adherence to medications [160], the existing data are insufficient to fully support the beneficial effects of statin therapy in the prevention and treatment of bone health.

High serum cholesterol also promotes the development of osteoarthritis [141]. Studies have shown that patients with osteoarthritis have high levels of crystals and cholesterol in their synovial fluid [161]. APOE mice chronically fed a high-cholesterol diet developed more severe osteoarthritis (OA) in a dose-dependent manner than did mice fed a normal diet [162]. Furthermore, a recent study revealed that cholesterol and its metabolites increase the risk of osteoarthritis by up-regulating matrix-degrading enzymes in chondrocytes through the stimulation of retinoic acid receptor-related orphan receptor-α (RORα) [163]. For osteoarthritis, clinical trials and animal studies have also demonstrated the protective effect of statins in relieving symptoms [164]. However, other cholesterol-lowering drugs have not shown a protective effect, so the role of statins in osteoarthritis treatment is more likely to result from their anti-inflammatory effects [165]. Further research is needed to investigate the role of low-cholesterol strategies in osteoarthritis.

### Adipose-derived hormones and bone homeostasis

In addition to the typical influence of lipid molecules on bone homeostasis, numerous studies over the past 20 years have examined the effects of adipose-derived hormones on bone homeostasis. Adipokines secreted into adipose tissue, such as leptin and adiponectin, have endocrine and paracrine effects on the survival and function of osteoblasts and osteoclasts. In addition to leptin and adiponectin, some newly discovered adipose-derived factors, such as visfatin, nesfatin-1, apelin, and vaspin, may also affect bone homeostasis, and there may be other adipokines that are currently being discovered [3]. This review focuses on the 2 most representative adipokines that have been shown to influence bone homeostasis, namely, leptin and adiponectin. It is worth noting that research on their effects on bone homeostasis has only just begun in the past 20 years, and the currently available evidence, including the inconsistent results reflected by some preclinical studies and limited clinical studies, also reflects the complex effects of these adipokines on bone homeostasis.

#### Leptin

Leptin, a well-conserved nonglycosylated peptide, has a molecular weight of 16 kDa and consists of 146 amino acids. Leptin is produced by the leptin gene in adipose tissue [166]. Traditionally, leptin suppress appetite through the nervous system, reduces insulin sensitivity, inhibits fatty acid synthesis, and limits fat storage [167]. However, ongoing discoveries over the last 20 years have shown that leptin may regulate bone metabolism through central and peripheral regulation. The central mechanism proposes that leptin inhibits bone formation by stimulating the sympathetic nervous system (SNS) through the hypothalamus, and the peripheral mechanism involves leptin directly interacting with leptin receptors on osteogenic or osteoclastic progenitor cells, thereby affecting their activity and directing their differentiation.

**Fig. 4.**
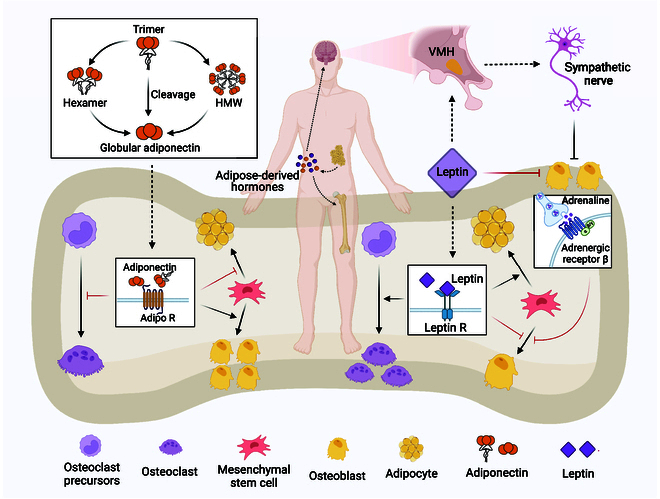
Regulation of bone homeostasis by adipokines. (1) The full-length adiponectin, which is composed of globular adiponectin and full-length adiponectin, can be cleaved to generate globular adiponectin. Adiponectin exerts its effects by binding to adiponectin receptors on the surface of bone cells. In the osteoblastic lineage, adiponectin signaling inhibits the adipogenic differentiation of BMSCs and promotes osteogenic differentiation. In the osteoclastic lineage, adiponectin directly or indirectly inhibits osteoclast activation. (2) Leptin regulates bone metabolism through 2 mechanisms: In the central nervous system, leptin increases sympathetic nerve activity, thereby inhibiting osteoblast activation. In peripheral tissues, leptin acts on leptin receptors in BMSCs, promoting adipogenic differentiation and inhibiting osteogenic differentiation; simultaneously, leptin enhances osteoclast activation.

Leptin and bone homeostasis: Osteoblasts: Initially, a series of experiments by Ducy and colleagues [168] revealed that in vivo, leptin inhibits bone formation, and mice in which leptin or leptin receptors are knocked out exhibit a high bone mass phenotype. Transplantation of normal adipose tissue to “fat-free” mice restores their skeletal phenotype, but transplantation of adipose tissue from leptin knockout mice cannot normalize the skeletal phenotype, supporting Ducy et al.’s view that leptin inhibits bone formation [169]. These results suggest that leptin produced by adipose tissue may suppress bone formation through multiple pathways. By genetically or pharmacologically ablating adrenergic signaling, which leads to leptin resistance and high bone mass, Takeda et al. [170] speculated that leptin may regulate bone metabolism through the SNS. Analysis of mice with defects in the β2-adrenergic receptor (Adrb2) confirmed that leptin primarily inhibits bone formation through the central nervous–sympathetic nervous system [170,171]. Specifically, leptin acts on ventromedial hypothalamus (VMH) nucleus neurons [172], which serve as a relay station to enhance SNS tone in bone. The SNS releases norepinephrine, which inhibits osteogenesis and bone formation by activating Adrb (Adrb2 in humans and Adrb1 in mice) on the surface of osteoblasts (Fig. 4).

Another possible mechanism is through its direct action on the surface receptors of stem cells, reversing the osteogenic differentiation of BMSCs toward adipogenic differentiation. BMSCs expressing leptin receptors are the main source of osteoblasts and adipocytes, which are usually quiescent. However, under external stimuli such as radiation or trauma, the activation of leptin receptors on BMSCs may lead to increased osteogenic, adipogenic, or chondrogenic differentiation [173]. Specific deletion of leptin receptors in BMSCs from the long bones of mice resulted in increased osteogenic differentiation and decreased adipogenic differentiation, with consistent results observed in vivo. The mineral deposition rate and bone formation rate of the long bones of the mice were significantly increased. Conversely, sustained elevation of the local leptin concentration in the skeleton led to up-regulation of adipogenic differentiation in BMSCs, with a decrease in the mineral attachment rate and bone formation rate of the long bones of mice, indicating that leptin receptor signaling promotes fat formation and inhibits osteogenic differentiation of BMSCs [174].

Osteoclasts: Consistent with the regulatory mechanisms governing osteoblasts, leptin may similarly modulate osteoclasts through 2 mechanisms mediated by the central nervous system and the peripheral neuroendocrine system. Primarily, leptin can activate a cascade of signaling pathways, including the Janus kinase 2 (JAK2)–signal transducer and activator of transcription 3 (STAT3), AMPK, and PI3K-AKT-FOXO1 pathways, via leptin receptors in the central nervous system, particularly in the hypothalamus. These pathways not only regulate energy metabolism but also influence bone metabolism by modulating the generation and function of osteoclasts [175]. For instance, studies have revealed that leptin inhibits AMPK signaling in the hypothalamus, thereby reducing osteoclastogenesis and activity and ultimately attenuating bone resorption [176,177].

Second, in peripheral tissues, leptin directly affects osteoclast behavior through autocrine and paracrine pathways. Leptin can directly act on osteoclasts and precursor cells, regulating their differentiation and function. For example, leptin can suppress the expression of RANKL in BMSCs and decrease osteoclast activation and generation by inhibiting RANKL signaling [178]. However, it is worth noting that Yue et al.’s [174] study also revealed that high concentrations of local leptin resulted in increased numbers of osteoclasts and significantly enhanced bone resorption in the distal metaphysis of the femur in mice, suggesting that leptin may also directly or indirectly up-regulate osteoclast differentiation and bone resorption.

Additionally, leptin may promote osteoclast apoptosis, reducing the number and activity of mature osteoclasts and thereby decreasing bone resorption. Several studies have explored the role of leptin in osteoclast apoptosis [179,180]. Leptin interacts with its receptors on osteoclasts, initiating signaling pathways that lead to apoptosis. For instance, it has been shown that leptin can induce apoptosis in osteoclasts through mechanisms involving the activation of proapoptotic pathways and the inhibition of anti-apoptotic proteins. This process helps to regulate the overall activity of osteoclasts, preventing excessive bone resorption, which can lead to conditions such as osteoporosis. One key mechanism by which leptin promotes osteoclast apoptosis is through the activation of caspases, which are crucial mediators of apoptosis [181].

Moreover, leptin may down-regulate the expression of markers associated with bone resorption, such as TRAP and acid phosphatase, further inhibiting the osteoclastic bone resorption capacity [178]. In postmenopausal women, correlations have been observed between serum leptin levels and markers of bone resorption, such as TRAP, acid phosphatase, and osteoclastic bone resorption capacity [182].

Osteocytes: In the regulation of bone homeostasis, leptin signaling in mature osteocytes shares many similarities with osteoblasts, regulating bone metabolism through both central and peripheral mechanisms [183]. On the one hand, leptin acts via the central nervous system, particularly the hypothalamus, to influence bone metabolism. It modulates the activity of the SNS, thereby affecting bone remodeling. Leptin binds to receptors in the hypothalamus, stimulating SNS activity, which leads to the release of norepinephrine. Norepinephrine acts on β-adrenergic receptors on bone cells, influencing bone resorption and formation. On the other hand, peripherally, leptin directly acts on bone cells, with its receptor expressed on osteocytes. The direct action of leptin regulates several intracellular signaling pathways: Leptin binding to specific receptor activates the JAK signal transducer and STAT pathway [184], which is crucial for transmitting leptin signals into the cell nucleus, leading to gene expression involved in cell survival, differentiation, and proliferation. Leptin also activates the PI3K/Akt pathway, playing a role in promoting osteocyte survival and preventing apoptosis [185]. This pathway enhances osteocytes’ resistance to various stressors, thus maintaining skeletal integrity. Additionally, the Akt signaling pathway helps regulate the expression of bone cell-specific genes, including the key bone formation regulator, sclerostin (SCL). The mitogen-activated protein kinase (MAPK) pathway is another crucial signaling cascade activated by leptin in bone cells. MAPK signaling is involved in regulating cellular responses to mechanical stress, which is essential for the mechanosensing functions of osteocytes [186]. Through this pathway, leptin helps bone cells respond to mechanical loads, promoting bone formation and remodeling. In summary, leptin plays a multifaceted role in bone cell signal transduction through central and peripheral mechanisms. By activating intracellular pathways such as JAK/STAT, PI3K/Akt, and MAPK, leptin influences osteocyte survival, mechanosensing capabilities, and the regulation of bone remodeling processes. Understanding these mechanisms provides valuable insights into the complex interactions between energy metabolism and skeletal health.

Clinical studies and applications of leptin: Currently, clinical studies on leptin are limited to investigating the correlation between serum leptin levels and various bone metabolism indices. Higher serum leptin concentrations and a positive correlation with body mass index (BMI) have been confirmed in obese postmenopausal women with osteoporosis [187,188]. Moreover, BMD increases with increasing BMI [188]. A meta-analysis of 45 studies showed a positive correlation between leptin levels and BMD and bone mineral content in postmenopausal women, indicating that leptin may be a bone tissue protective factor in postmenopausal women. However, these statistically significant differences may need to be interpreted with caution due to confounding factors such as leptin source and detection methods. A recent meta-analysis presented opposite results, indicating that serum leptin levels in postmenopausal women with decreased bone density tended to decrease compared with those in the control group with normal bone density [187].

It should be noted that there are numerous factors influencing the changes in bone mass in postmenopausal women, particularly after menopause, where the redistribution of various hormones and related receptors can lead to alterations in adipose tissue storage, serum leptin levels, and bone mass [189]; for instance, follicle-stimulating hormone (FSH) is considered to regulate gonadal functions and substantially participates in fat and bone metabolism [190]. Within the pituitary–gonadal axis, FSH stimulates osteoclast activation, while thyroid stimulating hormone (TSH) inhibits osteoclast bone resorption. Adrenocorticotropic hormone promotes osteoblast activation and bone formation, whereas leptin-mediated SNS activation may negatively regulate bone remodeling [191]. The role of FSH in bone loss and fat increase may be more significant than that of leptin [192]. Therefore, when discussing the correlation between serum leptin levels and bone mass, it may be challenging to provide a definitive answer based on the current state of research.

Another important clinical application of studying the correlation between leptin and bone density is predicting the risk of osteoporosis, especially postmenopausal osteoporotic fractures. Adding other indicators may improve the accuracy of fracture risk prediction compared to using leptin alone [111]. Moreover, it is worth noting that the effect of serum leptin levels on osteoporotic fractures may vary by bone type and location. Mohiti-Ardekani et al. [193] found no significant correlation between serum leptin levels and BMD in either osteoporotic or non-osteoporotic groups when analyzing fasting serum leptin levels and lumbar spine BMD. In contrast, Meng et al.’s [183] Mendelian randomization study showed that higher serum circulating leptin levels were associated with decreased lumbar spine BMD but not with decreased femoral neck, forearm, or whole-body BMD.

In addition, leptin influences bone metabolism via the SNS, indicating that Adrb antagonists, which are commonly utilized for treating cardiovascular conditions, might have therapeutic benefits for osteoporosis. Recent attention has been given to how Adrb antagonists enhance bone density and lower the risk of osteoporotic fractures, garnering significant interest among medical professionals. Studies have demonstrated that Adrb antagonists can improve both bone density and trabecular architecture in postmenopausal women, particularly in critical areas such as the femoral neck [194]. Their use alone or in combination with other antihypertensive drugs decreases the risk of osteoporotic fractures to varying degrees [195–197]. Notably, the effect of β-blockers on bone homeostasis varies depending on the subtype of the receptor. In rodents, β-blockers primarily act on Adrb2, while in humans, the primary target receptor for β-blockers is Adrb1 [198]. Therefore, selective β1-blockers may be more specific and effective in preventing and treating osteoporosis [198,199].

Multiple clinical studies have indicated that plasma, serum, and synovial fibroblast leptin levels affect OA progression [200]. Ait Eldjoudi et al. [200] and Min et al. [201] observed that leptin expression was greater in knee OA patients than in controls. Moreover, in the isolated articular cartilage of OA patients, leptin levels in synovial fibroblasts increase more markedly than do serum leptin levels, and both are positively correlated with BMI [200]. In addition, studies have shown that leptin up-regulates matrix metalloproteinase (MMP) production in articular cartilage involved in the progression of OA [202]. Body weight is an important risk factor for the incidence and progression of knee OA. Leptin levels are significantly greater in obese OA patients than in non-obese OA patients, demonstrating that leptin mediates the relationship between OA and obesity [203].

Moreover, leptin is also involved in the pathogenesis of rheumatoid arthritis (RA) [200]. However, clinical studies comparing leptin concentrations in serum or synovial fibroblasts from RA patients and healthy individuals have shown conflicting results. It has been reported that synovial fibroblast leptin concentrations are greater in patients with erosive RA than in those with nonerosive RA [204]. In addition, leptin has been shown to have strong proinflammatory effects in a mouse model of RA, and the use of leptin receptor antagonists has been shown to reduce the severity of the disease [205]. Numerous studies have demonstrated the ability of leptin to promote cartilage metabolism as well as systemic and local joint inflammatory responses, which provides further evidence of the key role of leptin in the pathogenesis and progression of RA and OA. However, some of the controversial results in published clinical studies may be due to the heterogeneity of clinical confounders in different study populations.

In summary, the effect of leptin on bone homeostasis is complex. The following highlights may be helpful in furthering our understanding of the effects of leptin on bone homeostasis: (a) Leptin may regulate bone homeostasis through both central and peripheral pathways, with central regulation involving the hypothalamus as a relay and acting through the SNS to modulate the differentiation and activity of bone cells; peripheral regulation occurs directly through leptin receptors on MSCs and/or osteoblasts. (b) The presence of multiple leptin receptor subtypes can lead to different cellular biological effects [206]. (c) Leptin may have different effects on different bone sites and types. (d) When exerting its effects in vivo, leptin faces a complex internal environment influenced by external factors. These factors can also affect the expression of other hormones, such as insulin and osteocalcin (OCN), thereby affecting bone metabolism differently [207]. In the future, further research on leptin and its receptors may provide potential therapeutic options for preventing and treating osteoporosis.

#### Adiponectin

Although there are differences in the reported concentrations of adiponectin in serum, adiponectin is almost certainly the most abundant adipokine secreted by adipocytes in circulation [208–210]. Adiponectin was originally identified in adipose tissue [211], but it was subsequently found that myocytes [212], osteoblasts [213], and other tissue cells can also produce adiponectin. However, adipose tissue and adipocytes, especially white adipose tissue and bone marrow adipose tissue, remain the main sources of serum adiponectin. The types of adiponectin in serum include full-length adiponectin and globular adiponectin. Full-length adiponectin mostly exists in oligomeric forms, including trimeric (low molecular weight), hexameric (medium molecular weight), and 12- or 18-mer (high molecular weight) forms. Globular adiponectins (gAds) are essentially 3 C-terminal globular domains that are tightly bound together by hydrophobic interactions within the trimeric core (Fig. 4). It can be generated by the cleavage of high-molecular-weight adiponectin by elastase from leukocytes [29,214]; therefore, high-molecular-weight adiponectin may serve as a reservoir of globular adiponectin [215]. The existence of various oligomeric structures of adiponectin endows it with multifaceted activities, and different oligomers act on different target tissues with distinct biological effects.

Adiponectin and bone homeostasis: Osteoblasts: In most preclinical studies, adiponectin signaling has been recognized to play a positive role in bone metabolism, as it is believed to promote osteoblast differentiation of BMSCs and preosteoblasts while inhibiting the activation and maturation of osteoclasts. Osteoblasts serve as direct targets of adiponectin, and adiponectin dose- and time-dependently promotes osteoblast proliferation and differentiation [216]. In vitro, adiponectin has been shown to enhance osteoblast differentiation and mineralization. This occurs through the PI3K/Akt signaling pathway, which is pivotal for cell survival and growth. For instance, adiponectin treatment increases oncostatin M expression [217] in osteoblasts via the PI3K/Akt pathway, further promoting osteogenic activities. The PI3K/Akt pathway is one of the most significant pathways facilitating the up-regulation of osteoblast differentiation markers and enhancing mineralization. This pathway activation is crucial for the survival and proliferation of osteoblasts [218].

Additionally, adiponectin influences the NF-κB pathway, which is known for its role in inflammatory responses and bone resorption processes. By modulating NF-κB activity, adiponectin can potentially reduce bone resorption and promote bone formation. Lee et al. [218] indicated that adiponectin enhances the expression of genes and proteins critical for osteoblast differentiation, such as RUNX2 and OCN, through its interaction with these signaling pathways. In addition, adiponectin has been shown to inhibit apoptosis in osteoblasts by modulating several intracellular signaling pathways, such as the AMPK and MAPK pathways. This inhibition of apoptosis leads to increased bone formation and reduced bone loss, thereby maintaining bone density [219,220]. In the study by Wu et al. [220], the in vitro addition of adiponectin to human periodontal ligament cell culture medium enhanced the expression of osteogenic-related genes, up-regulated the formation of mineralized nodules, and promoted osteogenic differentiation. In vivo, experiments in a number of animals have also verified the role of lipocalin in promoting osteoblast differentiation, and mice with adiponectin deficiency show a tendency for skull osteoblasts (primary osteoblasts) to differentiate into adipocytes rather than osteoblasts [221].

Osteoclasts: In the osteoclast lineage, primary BMMs from mice showed significant inhibition of proliferation and osteoclast differentiation with increasing concentrations of exogenous adiponectin during in vitro culture [222]. Similarly, adiponectin inhibited RANKL-induced osteoclastogenesis in mouse RAW 264.7 cells and disrupted the formation of the F-actin ring through adiponectin receptor (adipoR), including adipoR1 and adipoR2 [213]. The signaling pathways involved may include the AMPK, PI3K/protein kinase B (PKB), MAPK, STAT3, mTOR, NF-κB, and PPARα pathways [223]. Silencing adipoR with small interfering RNA (siRNA) eliminates adiponectin-induced cell proliferation and activation [216]. There is extensive evidence from animal models supporting the positive role of adiponectin signaling in regulating bone homeostasis. In a rat tibial fracture model, local injection of adiponectin into the fracture site led to increased bone formation marker and adipoR1 expression, resulting in significantly accelerated bone healing in rats [224]. Adenovirus-mediated gene transfer of adiponectin improved peri-implant bone integration in ovariectomy-induced osteoporotic rats [225]. Pharmacological activation of adiponectin signaling reduced bone loss in a mouse model of inflammatory bone loss, supporting the role of adipoR1 activation in promoting osteogenesis and reducing adiposity and osteoclastogenesis [226,227] (Fig. 4).

Osteocytes: Adiponectin also plays a significant role in the regulation of bone metabolism by influencing the signaling pathways in mature osteocytes. Adiponectin binds to its receptors, AdipoR1 and AdipoR2, which are expressed on osteocytes, activating several intracellular signaling pathways and impacting osteocyte function and bone homeostasis [228]. One of the primary pathways activated by adiponectin is the AMPK pathway. AMPK is a key energy sensor that maintains cellular energy homeostasis. When adiponectin binds to its receptors on osteocytes, it activates AMPK, which subsequently enhances osteocyte survival and function [229]. Activation of AMPK promotes the expression of genes involved in autophagy and reduces oxidative stress, which is critical for maintaining osteocyte viability and preventing apoptosis under metabolic stress conditions [229]. Another crucial pathway influenced by adiponectin is the PPAR signaling pathway. Adiponectin enhances the activity of PPARγ, a nuclear receptor that regulates the expression of genes involved in lipid metabolism and inflammation [230]. Through PPARγ activation, adiponectin reduces the production of pro-inflammatory cytokines and increases the expression of anti-inflammatory molecules. This anti-inflammatory effect helps to protect osteocytes from inflammation-induced damage, thereby supporting bone remodeling and maintenance. Moreover, adiponectin modulates the MAPK pathway, which is involved in the response to mechanical stress. By activating the MAPK pathway, adiponectin enhances the osteocytes’ ability to sense and respond to mechanical loading, a critical function for bone strength and adaptation. This pathway contributes to the regulation of bone formation and resorption, ensuring proper bone remodeling in response to mechanical stimuli [231,232]. Additionally, adiponectin has been shown to influence the production of SCL, a protein secreted by osteocytes that inhibits bone formation [233]. By down-regulating SCL expression, adiponectin promotes osteoblast activity and bone formation. This regulatory mechanism is vital for balancing bone resorption and formation, contributing to overall bone health. In summary, adiponectin plays a multifaceted role in osteocyte signaling transduction, affecting bone metabolism through the activation of AMPK, PPAR, and MAPK pathways. These pathways collectively enhance osteocyte survival, reduce inflammation, improve mechanical sensing, and regulate bone remodeling processes.

Clinical studies and applications of adiponectin: However, inconsistent with preclinical findings, most clinical observations over the past decade show that adiponectin has a negative effect on bone homeostasis [234–236]. Several meta-analyses have identified adiponectin as the most relevant adipokine negatively associated with postmenopausal osteoporosis [187,237]. Elevated levels of adiponectin may be associated with increased fracture risk in postmenopausal women [235] and suggest a high risk of vertebral fractures in men [237]. Clearly, these clinical observational studies are highly heterogeneous, and eliminating confounding factors such as sex, age, BMI, and disease status could lead to different conclusions.

Most studies on the pathogenesis of OA and RA suggest that adiponectin is a proinflammatory protein that plays an important role in the incidence and progression of OA and RA [238]. A meta-analysis by Tang et al. [239] indicated that the concentrations of adiponectin were greater in patients with OA than in healthy individuals and were also locally elevated in the synovium. In vitro experiments on synovial fibroblasts have shown that adiponectin triggers the generation of pro-MMP-1 and interleukin-6 (IL-6), which are key mediators of disruptive arthritis [238]. In addition, increased secretion of MMP-1, MMP-3 and MMP-13 was found in primary chondrocytes from OA patients after treatment with adiponectin [240]. Similarly, a number of studies have shown that adiponectin can activate the secretion of inflammatory mediators such as prostaglandin E2, IL-6, IL-8, MMP-1, and MMP-13 from synovial fibroblasts by stimulating adipoRs [238]. One hypothesis for the treatment of arthritis is to regulate adiponectin to reduce its adverse effects and promote its beneficial effects. However, the response of target cells to adiponectin stimulation may differ from patient to patient; therefore, the response to therapeutic measures may also differ. Therefore, further exploration of the mechanism of action of adiponectin may provide ideas for the development of personalized therapies based on the modulation of adiponectin activity.

Although conflicting conclusions exist between animal models and human studies, changing serum adiponectin levels or inducing adiponectin signal transduction through drugs may indeed have an effect on altering bone metabolism. Gong et al. [224] extracted highly purified and recombinant human adiponectin and used it for in vivo studies of bone marrow injection to accelerate bone healing, which may be a very meaningful first exploration of the drug effects and future clinical translation potential of human recombinant adiponectin. Further preclinical studies may be needed to clarify the specific mechanisms by which different adiponectin subtypes affect the biological state of bone cells and how the expression status of bone cell adipoR determines the response to adiponectin under various physiological or pathophysiological conditions. Moreover, large-scale, high-quality clinical studies could be highly beneficial for further clarifying the true role of adiponectin in bone. More importantly, recombinant human adiponectin and small molecule reagents targeting adipoR may provide potential clues for drug therapy for bone metabolic diseases.

## Bone Homeostasis Affects Lipid Metabolism

Traditionally, bone is viewed as a structural organ that provides mechanical support, protects soft tissues, and enables movement. As one of the largest organ systems in the body, accounting for approximately 15% of total body weight, the function of the skeleton extends beyond structural support. Bone homeostasis affects lipid metabolism in at least 2 distinct ways. First, from a macroscopic perspective, the metabolic competition theory posits that when the energy demand of bone tissue increases, it will consume a large amount of lipids from circulation to meet its metabolic needs, leading to changes in the overall lipid metabolism state. Second, from a microscopic perspective, skeletal cells secrete osteogenic cytokines, which have profound effects on fat metabolism through paracrine or endocrine mechanisms.

### Competition between bone formation and lipid storage

The skeletal system and its bone marrow components require substantial energy daily to regulate hematopoiesis and renewal, and to maintain bone mass. In adults, the skeleton experiences daily microfractures that require immediate repair. The fundamental process of bone homeostasis involves the active removal of bone by osteoclasts, followed by the replenishment of new bone by osteoblasts [241,242]. This closely coordinated process requires a significant amount of energy. As mentioned earlier, lipid metabolism is an crucial source of energy for bone remodeling. Considering the contradiction between the “energy demand” of bone remodeling and the “energy storage” of lipids, it can be inferred that changes in bone homeostasis will inevitably lead to changes in overall lipid metabolism [243]. When bone remodeling is activated, osteoblasts and osteoclasts rapidly obtain free fatty acids, triglycerides, cholesterol, or lipoprotein particles from the circulation to meet the increased energy demand of bone cells, which can be further metabolized through lipolysis and β-oxidation. These procedures lead to decreased lipid accumulation in peripheral cells and tissues, such as adipocytes in adipose tissue, hepatocytes in the liver, and myocytes in muscle tissue. Conversely, when bone remodeling slows, the energy demand of bone tissue decreases, resulting in a relative decrease in the availability of lipids and lipid substrates from circulation, leading to increased lipid storage in peripheral tissues.

The following evidence may support the theory of bone-lipid metabolic competition: First, the blood circulation in bone tissue is very rich, with bone receiving approximately ^1^/_10_ of the total cardiac output, ensuring that bone can obtain a large amount of lipids from circulation [244]. Niemeier et al. [245] have shown that osteoblasts and bones are second only to the liver in their uptake of circulating lipoproteins and free fatty acids. Second, animal models have shown that the development of high bone mass or low bone mass phenotypes is accompanied by changes in fat storage and metabolism. By genetically manipulating the expression of SCL in mice, there is a mutual “antagonistic” relationship between bone tissue formation and fat tissue deposition in mice [246]. Specific disruption of CPT-2 in osteoblasts and osteocytes in mice resulted in severe bone formation impairment, with significant lipid abnormalities due to obstruction of fatty acid utilization in osteoblasts and osteocytes. This suggests that when lipid utilization in bone cells decreases, the organism undergoes a redistribution of energy resources [35,37]. In addition, some clinical observational studies may also support the existence of a “competition” mechanism between bone homeostasis and lipid metabolism. Osteoporotic patients receiving teriparatide treatment showed improvements in bone density accompanied by decreases in triglycerides, weight, and BMI, indicating enhanced fat decomposition and utilization during bone formation [247]. A comparison of bone tissues extracted from patients with osteoporotic fractures, patients with osteoarthritis, and healthy autopsies revealed reduced bone formation. Meanwhile, key enzymes related to triglyceride metabolism, such as lipoprotein lipase and hormone-sensitive lipase, were expressed at lower levels, indicating reduced bone formation and decreased triglyceride breakdown and metabolism in bone tissue [248]. Based on the above evidence, a metabolically active skeletal system helps eliminate the adverse effects of lipid accumulation in other tissues and organs of the body; in other words, healthier bone function leads to better lipid metabolism. From a clinical pharmacotherapy perspective, improving the state of skeletal metabolism is a potential option for optimizing the lipid profile and reducing inappropriate fat accumulation.

### Bone-derived cytokines and lipid metabolism

Recent research has increased interest in cytokines derived from bone tissue, highlighting their role as endocrine or paracrine organs. Bone secretes numerous cytokines, with some already identified and others under investigation. These cytokines can significantly influence the metabolism of glucose, lipids, proteins, and inorganic salts across various tissues, organs, or systems. This review concentrates on 3 bone-derived cytokines closely linked to lipid metabolism: OCN, lipocalin-2 (LCN-2), and SCL.

#### Osteocalcin

OCN, the most prevalent noncollagenous protein in the bone matrix, is primarily produced by osteoblasts. It comprises 46 to 50 amino acids and undergoes posttranslational modifications, including vitamin K-dependent gamma carboxylation. The undercarboxylated form of OCN (Un-OCN) functions as an endocrine hormone in mammals, exhibiting a broad spectrum of biological effects. Un-OCN is known to enhance insulin sensitivity and glucose regulation, modulate lipid metabolism, boost male fertility, support memory and learning, increase exercise capacity, and play a role in acute stress responses. The endocrine functions of Un-OCN are attributed to its interaction with receptors such as GPR158 in the central nervous system and GPRC6A in peripheral tissues.

**Fig. 5.**
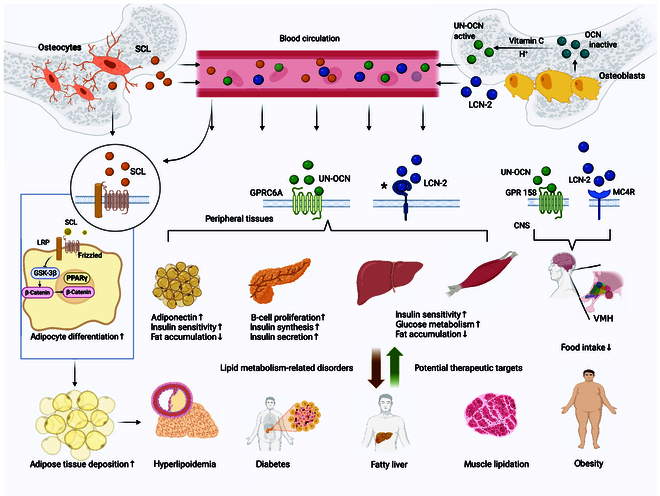
Regulatory effects of bone-derived cytokines on lipid metabolism. (1) Mature osteoblast are the primary source of sclerostin (SCL), while osteocalcin (OCN) and lipocalin-2 (LCN-2) are secreted by osteoblasts, with undercarboxylated OCN (Un-OCN) being the active form of OCN. Bone-derived cytokines exert their effects through paracrine or endocrine pathways. (2) In peripheral tissues, Un-OCN and LCN-2 signaling promotes the proliferation, insulin synthesis, and secretion of pancreatic β cells, enhancing peripheral tissue sensitivity to insulin and increasing glucose metabolism, directly or indirectly leading to increased fat breakdown and utilization, resulting in reduced fat accumulation. On the other hand, SCL up-regulates the typical Wnt/β-catenin signaling pathway and activates PPARγ, promoting adipocyte differentiation and increasing adipocyte size and number, leading to systemic accumulation of subcutaneous and visceral fat. (3) In the central nervous system, the binding of Un-OCN to hypothalamic neurons through GPR158 influences anxiety and stress behavior and indirectly alters blood glucose and lipid metabolism. Conversely, the binding of LCN-2 to hypothalamic neurons through MC4R reduces food intake, decreasing lipid accumulation and obesity.

Originally, research on OCN primarily focused on its role in glucose metabolism, so it is unsurprising that alterations in glucose processing could indirectly influence lipid metabolism and adiposity. Un-OCN has been shown to suppress the expression of genes such as sterol regulatory element-binding protein 1c and carbohydrate response element-binding protein in pancreatic islet β cells, thereby encouraging β cell proliferation. Conversely, in non-islet tissues, Un-OCN enhances glucose utilization and boosts cellular insulin sensitivity [249]. Moreover, Un-OCN can mitigate endoplasmic reticulum stress by activating the PI3K/Akt/NF-κB signaling pathway, thereby ameliorating insulin resistance in adipocytes. This leads to increased glucose utilization and decreased fat accumulation in cells [250]. In addition to indirectly affecting lipid and energy metabolism by modifying tissue cell sensitivity to insulin, Un-OCN also directly impacts lipid metabolism. A study by Otani et al. [251] demonstrated that Un-OCN up-regulates the expression of lipocalin and PPARγ in adipocytes. Subsequent in vivo experiments indicated that intermittent oral administration of Un-OCN reduced adipocyte volume in mice. These researchers also discovered that high doses of Un-OCN suppressed lipocalin and PPARγ expression in adipocytes, initiating necroptosis by enhancing FasL expression on adipocyte plasma membranes and activating Fas signaling in neighboring cells [252]. Additionally, Un-OCN plays a role in thermogenesis in brown adipocytes. Li et al. [253] used an in vitro model to confirm the specific function of osteocalcin in activating thermogenic genes in brown adipocytes (Fig. 5).

The regulation of lipid metabolism by Un-OCN in peripheral tissues occurs through GPRC6A on the cell membrane surface [254,255]. By conditionally deleting GPRC6A from male mouse hepatocytes, mouse liver cells were found to undergo steatosis, and cholesterol and triglycerides were deposited in hepatocytes [256]. Importantly, a recent study highlighted the effects of adipocyte-specific GPRC6A knockout in mice. These genetically modified animals, when subjected to a high-fat and high-sucrose diet, displayed significant increases in adipose tissue weight, adipocyte hypertrophy, and inflammation within the adipose tissue compared to their control counterparts. This finding underscores the critical role of the GPRC6A receptor in regulating adipose tissue responses and metabolic processes under diet-induced stress conditions [257]. In the central nervous system, Un-OCN has the ability to cross the blood–brain barrier and bind to the GPR158 receptor in the hippocampus. This interaction promotes the release of various neurotransmitters, subsequently influencing behaviors such as anxiety, cognition, and stress responses. This mechanism highlights the broader systemic effects of Un-OCN beyond its metabolic and bone-related functions, demonstrating its role in neural processes and behavioral regulation [258], as well as its indirect effects on glucose and lipid metabolism.

Overall, impaired Un-OCN mechanisms may lead to abnormal fat accumulation in peripheral organs and tissues such as the liver, skeletal muscle, and fat, which ultimately leads to metabolic syndrome. Several clinical observational studies have shown a correlation between reduced secretion of Un-OCN and disorders of lipid metabolism [259–261]. Zheng et al. [259] reported that decreased Un-OCN levels in children with osteogenesis imperfecta were accompanied by imbalances in glucose and fat metabolism, hyperglycemia, and obesity. Bao et al. [260] found that an increase in serum osteocalcin levels was accompanied by a decrease in triglyceride levels, insulin resistance index, and a reduction in visceral fat accumulation. The above findings suggest that serum Un-OCN may be useful for screening for metabolic diseases, and more importantly, regulating the secretion of Un-OCN from skeletal cells may be a potential solution for the treatment of lipid metabolism disorders and metabolic syndrome in the future.

#### Lipocalin 2

Researchers have recently worked to identify bone-derived cytokines other than OCN that affect energy metabolism. Studies have revealed that the specific ablation of osteoblasts in adult mice leads to hyperglycemia, impaired glucose tolerance, and reduced insulin sensitivity. Although supplementation with osteocalcin ameliorated glucose intolerance, it only partially restored insulin sensitivity and had no impact on gonadal fat weight or energy expenditure in osteoblast-depleted mice [262]. These observations imply that osteoblasts might secrete additional hormones or cytokines that enable bones to regulate energy metabolism. Rached et al. [263] found a substantial increase in LCN-2 expression in mice with specific knockdown of forkhead box protein O1 (FOXO1) in osteoblasts. A subsequent study showed that the application of FOXO1 siRNA down-regulated FOXO1 expression in rat osteoblasts, followed by the up-regulation of LCN-2 expression, which positively affected glucose metabolism [264]. These results provide compelling evidence for the role of the FOXO1/LCN-2 signaling pathway in osteoblasts in the regulation of energy metabolism. LCN-2, also known as neutrophil gelatinase-associated lipocalin, is a secreted glycoprotein initially identified as an adipokine [265]. Although it is produced by neutrophils and various tissues, such as the liver, bone marrow, uterus, prostate, stomach, lung, colon, and adipose tissue, expression profiling has shown that LCN-2 is expressed at least 10 times more abundantly in bone than in adipose tissue or any other tissue. This high expression level in bone highlights its potential role in bone physiology and systemic energy metabolism [266,267].

Overall, LCN-2 may regulate lipid metabolism in the following 3 ways. First, LCN-2 secreted by osteoblasts plays a crucial role in maintaining glucose homeostasis by inducing insulin secretion, which improves glucose tolerance and insulin sensitivity. These changes indirectly affect lipid metabolism. Experimental findings indicate that silencing LCN-2 expression exacerbates metabolic dysfunction in both genetically obese and diet-induced obese mice. Conversely, overexpression of LCN-2 aids in maintaining glucose tolerance, reduces dyslipidemia, and prevents hepatic lipid accumulation and steatosis [268]. Increasing circulating LCN-2 levels has also been shown to improve metabolic parameters and promote β cell function in models of β cell failure [269]. Conversely, global genetic knockout of LCN-2 in mice leads to accelerated weight gain and visceral fat accumulation over time compared to wild-type controls [270,271]. Interestingly, while complete LCN-2 knockout increases glucose tolerance, it does not alter insulin sensitivity. However, osteoblast-specific LCN-2 knockout mice displayed reduced glucose tolerance and impaired insulin sensitivity [267], suggesting that osteoblast-derived LCN-2 up-regulation serves as a protective mechanism that enhances glucose metabolism and reduces fat accumulation (Fig. 5). Second, LCN-2 can cross the blood–brain barrier and interact with melanocortin 4 receptors (MC4Rs) in hypothalamic paraventricular and ventral medial neurons, activating pathways that induce anorexia [267,272]. Radiolabeled recombinant human LCN-2 (rh-LCN-2) administered to primates localized to the hypothalamus, as confirmed by brain positron emission tomography (PET) scans and radiographic autoradiography. Treatment with rh-LCN-2 also led to reduced food intake in these animals. This role of LCN-2 as a satiety factor underlines its significance in regulating feeding behaviors [272]. In addition, LCN-2 is crucial for the thermogenesis of brown adipose tissue [268,273–275]. A deficiency in LCN-2 leads to reduced thermogenic activity in brown adipocytes, characterized by decreased levels of brown adipose tissue markers and diminished mitochondrial oxidative phosphorylation [273]. In contrast, LCN-2 overexpression in mice prevents age-related decreases in adipose tissue thermogenesis and disruptions in adipose metabolism [268]. These varied roles of LCN-2 in metabolic regulation highlight its potential as a target for therapeutic interventions in metabolic disorders.

Some animal models of diseases have revealed a link between lipid metabolism imbalance and LCN-2 levels. LCN-2 is robustly up-regulated in mouse models of pancreatic cancer, and LCN-2 levels are associated with adiposity, lean body mass depletion, tumor cachexia, and mortality. Mice with genetic deletion of LCN-2 exhibit increased inguinal adiposity and body weight [276]. Recent clinical observations revealed that human circulating LCN-2 was negatively correlated with serum triglycerides and positively correlated with HDL cholesterol [277]. The above evidence suggests that LCN-2 may be a meaningful serological indicator of lipid metabolism disorders and a new target for improving fat metabolism and treating obesity.

#### Sclerostin

SCL is a glycoprotein secreted by mature osteocytes, encoded by the sclerosteosis (SOST) gene. Although SCL is present in various tissues and organs, it is produced in particularly high quantities in bone tissue [278]. In recent years, growing evidence from in vitro studies, animal experiments, and clinical trials has demonstrated that SCL plays a significant role in regulating lipid metabolism.

In vitro studies have shown that recombinant SCL induces adipocyte differentiation [279–281]. The addition of SCL to conditioned medium significantly enhances the adipogenic differentiation of 3T3-L1 preadipocytes and primary BMSCs [280,281]. Mechanistically, the pro-lipidemic effect of SCL is due to the inhibition of the Wnt signaling pathway and a subsequent increase in PPARγ gene expression [280] (Fig. 5). More specifically, SCL binds to the primary adipocyte membrane surface GPR Lrp4/5/6 and reduces typical β-linked protein signaling, resulting in enhanced lipogenic differentiation [282]. In particular, Lrp4 plays an important role in enhancing the differentiation of adipocytes in vitro [282,283]. In vivo experiments revealed that SCL plays a role in facilitating adipogenesis. Studies involving SOST knockout mice, as well as mice treated with SCL-neutralizing antibodies, revealed a significant reduction in whole-body adiposity [246]. Additionally, in the white adipose tissue of SOST knockout mice, not only was there a general decrease in fat accumulation, but the individual adipocytes were also smaller. In contrast, mice that overexpress SCL by adenovirus [246] or are additionally injected with recombinant SCL [284] exhibited an increase in whole-body adiposity, an increase in the weight of a single fat pad, and an increase in the size of individual adipocytes, while the expression of Wnt target genes appeared to be down-regulated.

Clinical studies have provided evidence that serum SCL levels are positively associated with body fat mass and lipid profile markers. For example, patients with congenital generalized lipodystrophy, who typically struggle with lipid storage in adipocytes and exhibit low body fat, have been found to have elevated serum SCL levels [285]. In elderly individuals, serum SCL is positively correlated with total adiposity and BMI in both men and women and specifically correlated with vertebral bone marrow fat content in men [286]. Among postmenopausal women, higher serum SCL levels have been linked to increased body weight, BMI, and overall adiposity [287]. Additionally, recent research indicates that maternal SCL is positively correlated with fetal abdominal circumference and subcutaneous fat deposition, which contribute to higher birth weight [288]. Nevertheless, because the above clinical studies are cross-sectional/case–control studies and because of the technical uncertainties of current circulating SCL assay methods [289], these works need to be interpreted with caution; however, the above evidence suggests that there is indeed an overall positive correlation between SCL and adipogenesis. Notably, before SCL can be used in clinical applications, the following issues may need to be considered: first, whether there are coordinated or antagonistic effects of other signaling molecules on the regulation of adipocyte differentiation and adipose tissue formation by SCL, and second, whether the physiological concentrations of SCL that regulate adipocyte behavior in vivo and in vitro are affected. In addition, the degree of importance of SCL relative to other endocrine and paracrine factors in disease needs to be further determined.

## Future Perspective

Drawing from the aforementioned findings, we offer a perspective on future research focuses and potential challenges in the realm of bone–lipid interactions. In the domain of basic research, leveraging advancements in mass spectrometry and chemical imaging techniques will significantly expand our understanding of how lipid metabolism impacts bone homeostasis. This approach holds promise for identifying novel lipid signals that enhance bone quality and promote human health. Delving into the intricacies of lipid signal transduction and regulatory mechanisms could unveil potential drug targets for bone metabolism disorders. However, the exact effects of bone-derived cytokines on whole-body metabolism remain uncertain. Their precise role in lipid metabolism remains ambiguous—whether they exert a decisive influence or merely function as a minor component within the complex internal environment of the human body. It is plausible that bone-derived cytokines play a supporting role in metabolic changes primarily governed by other hormones. In the future, large-scale genetic screening coupled with bioinformatics predictions may shed light on previously unknown regulators of bone-derived factors involved in lipid metabolism. This holistic approach could deepen our understanding of bone-lipid interactions and contribute to the development of targeted interventions for metabolic disorders with implications for bone health.

From a translational medicine standpoint, certain lipid-related molecules hold potential as reliable biomarkers for evaluating bone metabolism and predicting the risk of bone metabolic disorders, such as osteoporosis and osteoporotic fractures. Conversely, bone-derived factors may also serve as biomarkers for metabolic diseases, including lipid metabolism disorders. Although their current predictive value may seem limited, the primary bottleneck lies in the scarcity of comprehensive clinical studies, with future high-quality randomized controlled trials showing promising prospects. Further advancements in precision and efficacy related to lipid interventions have the potential to enhance bone metabolism and promote human health. Bone-derived hormones and their receptors could emerge as novel targets for addressing metabolic diseases and their associated complications. Existing evidence supports the notion that regulating the composition and proportion of various fatty acids in the diet, along with enhancing blood lipid profiles through statins, exogenous bone-derived hormone analogs, or small-molecule interventions targeting their receptors, can yield beneficial effects on bone homeostasis and lipid metabolism. Nevertheless, it is essential to acknowledge that most of the evidence comes from basic research and animal models, and there remains a relative scarcity of effective clinical trials. Additionally, the specific implementation plans for these interventions, such as determining the optimal ratio for fatty acid regulation, identifying appropriate dosages and durations for statin use, and establishing suitable concentration levels and administration methods for exogenous hormones, present challenging issues that warrant thorough investigation and resolution.

## Conclusion

Despite the remaining questions that require clarification, it is evident that a close metabolic relationship and intricate regulatory network exist between lipid metabolism and bone homeostasis. Interventions targeting lipid-related pathways significantly impact bone metabolism, with specific lipids and lipid-related molecules influencing osteoblast and osteoclast differentiation and function. Concurrently, alterations in bone homeostasis exert complex effects on lipid metabolism through mechanisms such as “energy competition” and “endocrine” signaling. Recognizing bone as a pivotal endocrine organ in the organism unveils further interactive information between bone and lipids. Further exploration in this direction is likely to yield valuable insights.
